# A novel LncRNA transcript, RBAT1, accelerates tumorigenesis through interacting with HNRNPL and cis-activating E2F3

**DOI:** 10.1186/s12943-020-01232-3

**Published:** 2020-07-15

**Authors:** Xiaoyu He, Peiwei Chai, Fang Li, Leilei Zhang, Chuandi Zhou, Xiaoling Yuan, Yongyun Li, Jie Yang, Yingxiu Luo, Shengfang Ge, He Zhang, Renbing Jia, Xianqun Fan

**Affiliations:** 1 Department of Ophthalmology, Ninth People’s Hospital, Shanghai JiaoTong University School of Medicine, Shanghai, China; 2 Shanghai Key Laboratory of Orbital Diseases and Ocular Oncology, Shanghai, China

## Abstract

**Background:**

Long non-coding RNAs (lncRNAs) have been identified as important epigenetic regulators that play critical roles in human cancers. However, the regulatory functions of lncRNAs in tumorigenesis remains to be elucidated. Here, we aimed to investigate the molecular mechanisms and potential clinical application of a novel lncRNA, retinoblastoma associated transcript-1 (RBAT1), in tumorigenesis.

**Methods:**

RBAT1 expression was determined by real-time PCR in both retinoblastoma (Rb) and bladder cancer (BCa) cell lines and clinical tissues. Chromatin isolation using RNA purification (ChIRP) assays were performed to identify RBAT1-interacting proteins. Patient-derived xenograft (PDX) retinoblastoma models were established to test the therapeutic potential of RBAT1-targeting GapmeRs.

**Results:**

Here, we found that RBAT1 expression was significantly higher in Rb and BCa tissues than that in adjacent tissues. Functional assays revealed that RBAT1 accelerated tumorigenesis both in vitro and in vivo*.* Mechanistically, RBAT1 recruited HNRNPL protein to E2F3 promoter, thereby activating E2F3 transcription. Therapeutically, GapmeR-mediated RBAT1 silencing significantly inhibited tumorigenesis in orthotopic xenograft retinoblastoma models derived from Rb cell lines and Rb primary cells.

**Conclusions:**

RBAT1 overexpression upregulates a known oncogene, E2F3, via directly recruiting HNPNPL to its promoter and cis-activating its expression. Our finding provides a novel mechanism of lncRNA biology and provides potential targets for diagnosis and treatment of Rb and BCa.

## Introduction

Long non-coding RNAs (lncRNAs) are defined as transcripts longer than 200 nt that lack protein coding potential [1]. LncRNAs are involved in a wide range of biological processes and can regulate gene expression in cis or in trans by diverse mechanisms [2], such as RNA degradation, chromatin remodeling, and histone modifications [3]. For example, lncRNA MEG3 interacts Jumonji and AT-Rich Interaction Domain Containing 2 (JARID2), an essential component of Polycomb Repressive Complex 2 (PRC2), to silence target genes during embryonic stem cell differentiation [4]. In addition, lncRNA XIST acts as an important inactivator of X chromosome in early human development [5]. Conclusively, the dynamic roles of lncRNAs have attracted increasing attention in the diversified biological processes.

Since lncRNAs are vital in the maintenance of homeostasis, mutations or aberrant expression of certain lncRNAs may also lead to the occurrence of various diseases, especially in cancer [6]. To date, aberrant lncRNA expression has been demonstrated in many types of cancers and leads to abnormal cell proliferation, migration and apoptosis [7, 8]. For example, by forming a complex with heterogeneous nuclear ribonucleoprotein L (HNRNPL), cytoplasmic lncRNA CASC9 regulates genes linked to AKT signaling in hepatocellular carcinoma [9]. LncSox4, which physically binds to STAT3 and recruits a transcription factor to the SOX4 promoter, leads to SOX4 transcription and liver cancer cell self-renewal [10]. Therefore, further exploration of the lncRNAs drivers in tumorigenesis is potentially interesting.

Notably, retinoblastoma, the most common primary intraocular malignancy in children, has also been proven to be associated with lncRNA dysregulation. Our previous study has demonstrated lncRNA GAU1 could recruit Transcription Elongation Factor A1 (TCEA1) to the promoter of Polypeptide N-Acetylgalactosaminyltransferase 8 (GALNT8), thereby upregulate an oncogene expression and accelerate tumorigenesis of retinoblastoma [11]. In addition, the upregulation of lncRNA PANDAR has also been revealed to be involved in cell growth and apoptosis in retinoblastoma cell lines [12]. However, our knowledge of lncRNAs in tumors, especially in Rb, remains limited. We thus aimed to identify the molecular mechanisms and potential clinical application of lncRNAs in retinoblastoma.

In this study, we revealed RBAT1 as a novel non-coding transcript that plays a critical role in cell proliferation in vitro and in vivo by gain-of-function and loss-of-function experiments. We found that RBAT1 is an important transcriptional accelerator that induces E2F3 gene expression in tumor cells. Mechanistically, we showed that RBAT1 recruits the HNRNPL protein to E2F3 promoter region, which activates E2F3 signaling to prime for tumor cell proliferation. Therefore, our study elucidates the potential role of RBAT1 in the development of Rb and BCa and unveils its molecular mechanism underlying tumor progression. Our findings suggest that RBAT1 could be a potential therapeutic target for Rb and BCa.

## Methods

### Cell culture

The retinoblastoma cell lines Y79, WERI-Rb-1 and bladder carcinoma cell line 5637 were purchased from American Type Culture Collection (ATCC) from 2010 to 2015. These cells grown in RPMI 1640 medium (Invitrogen, Carlsbad, CA, USA) supplemented with 10% fetal bovine serum (FBS; Gibco). The patient-derived retinoblastoma primary cells were grown in RPMI 1640 medium supplemented with 10% FBS. The human colorectal cancer cell lines HT-29, HCT 116 and the melanoma cell lines A375 are obtained from ATCC at the year of 2015. PIG1 was gifted from Prof. Martin Jager at the year of 2016. OM431 was a kind gift from Tumor Biology Laboratory of John Vane Science Centre at the year of 2014. These cells were grown in Dulbecco’s modified essential medium (DMEM; Gibco, Carlsbad, CA, USA) with 10% FBS. The control cells were grown in RPMI 1640 medium (for SV-HUC-1) or DMEM (for ARPE-19, NCM460, PIG1) supplemented with 10% FBS. All media were supplemented with 1% penicillin/streptomycin. The cultures were maintained in a humidified atmosphere at 37 °C with 5% CO_2_. These cells were characterized by short tandem repeat (STR) markers and were confirmed to be mycoplasma-free (last tested in June 2019).

### Tissue specimens

Retinoblastoma tissues and adjacent normal retinal tissues were collected from affected eyes during enucleation at the Department of Ophthalmology, Ninth People’s Hospital, Shanghai JiaoTong University School of Medicine. The retinoblastoma tissues were harvested from vitreous cavity and adjacent normal retinal tissues were detached from eyeball. Bladder cancer tissues and adjacent bladder tissues were collected during radical cystectomy procedures at the Department of Urology Surgery, Changhai Hospital, Second Military Medical University. The clinical stage of Rb patients were classified according to the International Retinoblastoma Staging System (IRSS) [13], and BCa patients were classified according to American Joint Committee on Cancer (AJCC) staging [14]. Fresh tissue samples were snap-frozen in liquid nitrogen and stored at − 80 °C. All samples were pathologically confirmed. Patient consent and approval from the Institutional Research Ethics Committee were obtained prior to surgery.

### GapmeR

Cells were transfected at 60–80% confluence with 20–50 nmol/L locked nucleic acid (LNA) GapmeR (Exiqon, Vedbaek, Denmark) targeting RBAT1 using Lipofectamine 2000 (Invitrogen, Carlsbad, CA, USA) according to manufacturer’s instructions. As control, scrambled LNA GapmeR was transfected. Forty-eight hours after transfection, the cells were harvested for further analysis. GapmeR sequences are listed in Supplementary Table 3.

### shRNA-expressing plasmid construction

The pGIPZ lentivirus vector (System Biosciences, USA) was used to generate short hairpin RNAs (shRNAs) against E2F3 and HNRNPL and a negative control. The shRNA sequences were obtained using PCR with Xho I-Mlu I sites. Then, the sequences were cloned into the Xho I-Mlu I sites in the pGIPZ lentivirus vector. shRNA sequences are listed in Supplementary Table 3.

### Lentivirus packaging

The packaging procedure was performed as previously reported [15]. Briefly, 293 T cells were cultured in DMEM (Gibco, USA) supplemented with 10% FBS and transfected with 3 μg plasmid of pGIPZ-shE2F3, 6 μg of PsPax and 3 μg of pMD2.D using Lipofectamine 2000 (Invitrogen, Carlsbad, CA). The medium was replaced with 5 ml of fresh medium after an overnight incubation. The virus-containing supernatants were collected at 48 and 72 h after transfection. Then, the solutions were mixed together. The viral supernatants were filtered and concentrated. Colony selection was performed by culturing the tumor cell lines with 3–6 μg/ml puromycin for 2–3 weeks. The colonies with GFP expression were selected for subsequent culture.

### RNA isolation and real-time PCR

Total RNA was extracted from cells using TRIzol reagent and reverse transcribed with a PrimeScript RT-PCR kit (Takara Biotechnology) according to the manufacturer’s instructions. Real-time PCR was performed using a standard SYBR Green PCR kit (Roche). The 2^-△△Ct^ method was used to calculate the relative expression levels, and GAPDH transcription levels were used as an internal control. All specific primers are listed in Supplementary Table 3.

### Western blotting analysis

Western blotting analysis was performed using anti-E2F3 (Abcam, USA), anti-HNRNPL (Abcam, USA), anti-CDC6 (Proteintech), anti-CCNA1 (Proteintech), anti-β-tubulin (Abcam, USA), anti-β-actin (Sigma-Aldrich) and anti-H3 (Abcam, USA) primary antibodies. The blots were then incubated with a secondary antibody (Cell Signaling Technology) and visualized using an Odyssey Infrared Imaging System (LI-COR, USA).

### Immunohistochemistry

For immunohistochemistry, Anti-Ki67 (Abcam, USA) antibodies were used to detect Ki67 expression in mouse tumors. Images were taken using an Olympus BX 51 microscope (Olympus Corporation, Japan).

### RNA fish

Briefly, tumor cell lines were seeded and fixed with 4% paraformaldehyde. Then, they were treated with 0.5% Triton followed by pre-hybridization. Overnight hybridization was performed with a 10 mM probe concentration. An RNA FISH kit was purchased from Ribo Bio (Guangzhou), and the experiment was performed according to the manufacturer’s instructions. The Cy3-labeled 18S, U6 and RBAT1 probes were synthesized and provided by Sangon Biotech (Shanghai). Images were taken with a confocal microscope (Zeiss). The probe sequences used for FISH are listed in Supplementary Table 3.

### Cytoplasmic and nuclear RNA isolation

Nuclear and cytoplasmic RNA were isolated using a nuclear/cytosol fractionation kit (Biovison) according to the manufacturer’s instructions. Real-time PCR was performed to detect RBAT1 expression levels in both the cytoplasm and nucleus.

### CCK8 assay

CCK8 (Cell Counting Kit-8) colorimetric assays were performed to detect cell viability. Cells were seeded in 96-well plates at a density of 2000–5000 cells/well, and 10 μl of CCK8 solution was added to each well (100 μl medium). After 4 h of incubation at 37 °C with 5% CO_2_, absorbance was measured at 450 nm in a microplate reader (Varioskan Flash; Thermo, USA). The experiment was performed on four consecutive days.

### Soft agar assay

For the soft agar tumor formation assays, 2 ml of 0.8% agar (Sigma-Aldrich, USA) in complete medium was spread in each of the 6-well plates. A total of 15,000 cells were suspended in 1.5 ml of complete medium containing 0.4% agar and seeded into each well. Then, 200 μl of complete medium was added to each of the 6-well plates every 4 days. The tumor cells were cultured in soft agar for 4–5 weeks. The colonies were washed with PBS and then fixed and stained with a 0.01% crystal violet solution. Images were captured by a scanner, and the number of colonies in each well was detected by ImageJ software.

### Cell cycle assay

For the cell cycle analysis, cells were harvested and fixed with 75% ice-cold ethanol. After treatment with RNase A, the cells were stained with 50 μl/ml PI and treated with 0.5% Triton PBS. Then, the cells were incubated with an anti-H3 antibody (Abcam, USA) at a 1:100 dilution. The prepared cells were analyzed by a FACSCalibur flow cytometer (BD Bioscience). All data were collected and processed using BD FACSuite analysis software.

### Rapid amplification of cDNA ends (RACE) assay

Total RNA was extracted using a TRIzol Plus RNA Purification Kit (Invitrogen). RACE PCR products were obtained using Platinum PCR Supermix High Fidelity (Invitrogen) and separated on a 1.5% agarose (Sigma) gel. The gel products were extracted using a gel extraction kit, cloned into a pGM-T vector and sequenced. The specific 3′ RACE and 5′ RACE primers are listed in Supplementary Table 3.

### Chromatin isolation by RNA purification (ChIRP)

ChIRP was performed using a Magna ChIRP RNA Interactome Kit (Millipore) according to the manufacturer’s instructions [16–18]. A 3′ end Biotin-TEG modified-DNA probe targeting RBAT1 was synthesized and provided by Sangon. Before crosslinking, both retinoblastoma and bladder cancer cells were grown in RPMI 1640 medium supplemented with 10% fetal bovine serum at 37 °C in a humidified 5% CO^2^ atmosphere. Cells were seeded at a density of 4 × 10^6^/T25 bottom (for Y79 and WERI-RB1) and of 2 × 10^6^/10 cm dish. Cell lysates were harvested after incubation of 48 h with a confluence of 75%. Cells were cross-linked with 1% formaldehyde and sonicated for the hybridization reaction. After the chromatin was sheared into 100–500 bp fragments, the cell lysates were incubated with the biotinylated DNA probe solution for 4 h at 37 °C. The binding complex was covered with streptavidin-conjugated magnet beads. DNA, RNA, and protein were finally eluted and purified from the magnet beads for real-time qPCR or mass spectrometry and western blotting analyses. An additional sample was digested with 15 μg/ml RNase for 10 min at room temperature before incubation with RBAT1 probes to serve as a negative control. The sequences of the probes are available in Supplementary Table 3.

### RNA immunoprecipitation (RIP)

A Magna RNA binding protein immunoprecipitation kit was purchased from Millipore, and the experiment was conducted according to the manufacturer’s instructions. In brief, a total of 5 × 10^7^ cells were harvested and lysed using RIP lysis buffer. HNRNPL was immunoprecipitated using an anti-HNRNPL specific antibody (Abcam, USA), and the retrieved RNA was subjected to real-time qPCR analysis. An anti-IgG antibody was used as a negative control. For the real-time qPCR analysis, U6 was used as a nonspecific control, and THRIL was used as a positive control.

### Chromatin immunoprecipitation (ChIP)

ChIP assays were performed using an EZ-Magna ChIP A/G kit (Millipore). The experiment was conducted according to the manufacturer’s instructions and as previously reported in a detailed protocol. Anti-HNRNPL was obtained from Abcam. Anti-IgG was used as a negative control, and anti-RNA polymerase-II was used as a positive control. Specific primers used for ChIP are listed in Supplementary Table 3.

### Luciferase assays

Cells were transfected with a pGL3-based vector containing E2F3 promoter fragments. The cells were harvested after 48 h for firefly/Renilla luciferase assays using the Dual-Luciferase Reporter Assay System (Promega) according to the manufacturer’s protocol. Firefly luciferase values were normalized to Renilla luciferase values to control for transfection efficiency.

### Microarray analysis

The Agilent SurePrint G3 Human Gene Expression v3 Microarray was used in this study to detect the expression of genes after RBAT1 knockdown. The arrays were scanned with an Agilent Scanner G2505C (Agilent Technologies), and the data were analyzed using Feature Extraction software (version 10.7.1.1, Agilent Technologies). The microarray data after RBAT1 silencing has been uploaded in National Omics Data Encyclopedia (NODE) database (accession number: OEP000762). Gene Ontology (GO) analysis were performed in http://www.geneontology.org/. Gene network analysis were performed using STRING database (https://string-db.org/).

### In vivo animal model experiments

A total of 3 × 10^5^ tumor cells (4 × 10^5^ cells for the patient-derived xenograft (PDX) model) were implanted on the retinas through intraocular injection to establish a stable orthotopic retinoblastoma model in BALB/c nude mice (male, 4–5 weeks old). At 9 days after tumor cell inoculation, 15 mg/kg GapmeR1, GapmeR2 or control GapmeR was injected intravenously every 3 days for up to 30 days. Then, the mice were euthanized, and tumor bearing eyeballs were removed, fixed in 4% paraformaldehyde and weighted. The PDX models of retinoblastoma were developed. Written informed consent was obtained from the patients. All experimental procedures were approved by the Institutional Animal Care and Use Committee of the Ninth People’s Hospital, Shanghai JiaoTong University School of Medicine.

### Statistical analysis

Experiments were performed in triplicate while indicated, and the data are presented as the mean ± SD. *P* values were calculated by Student’s t-test. Survival plots were generated by Kaplan-Meier curve, and d P values were calculated by the log-rank test.

## Results

### The novel RBAT1 lncRNA is highly expressed in the nucleus of Rb and BCa

**Fig. 1:**
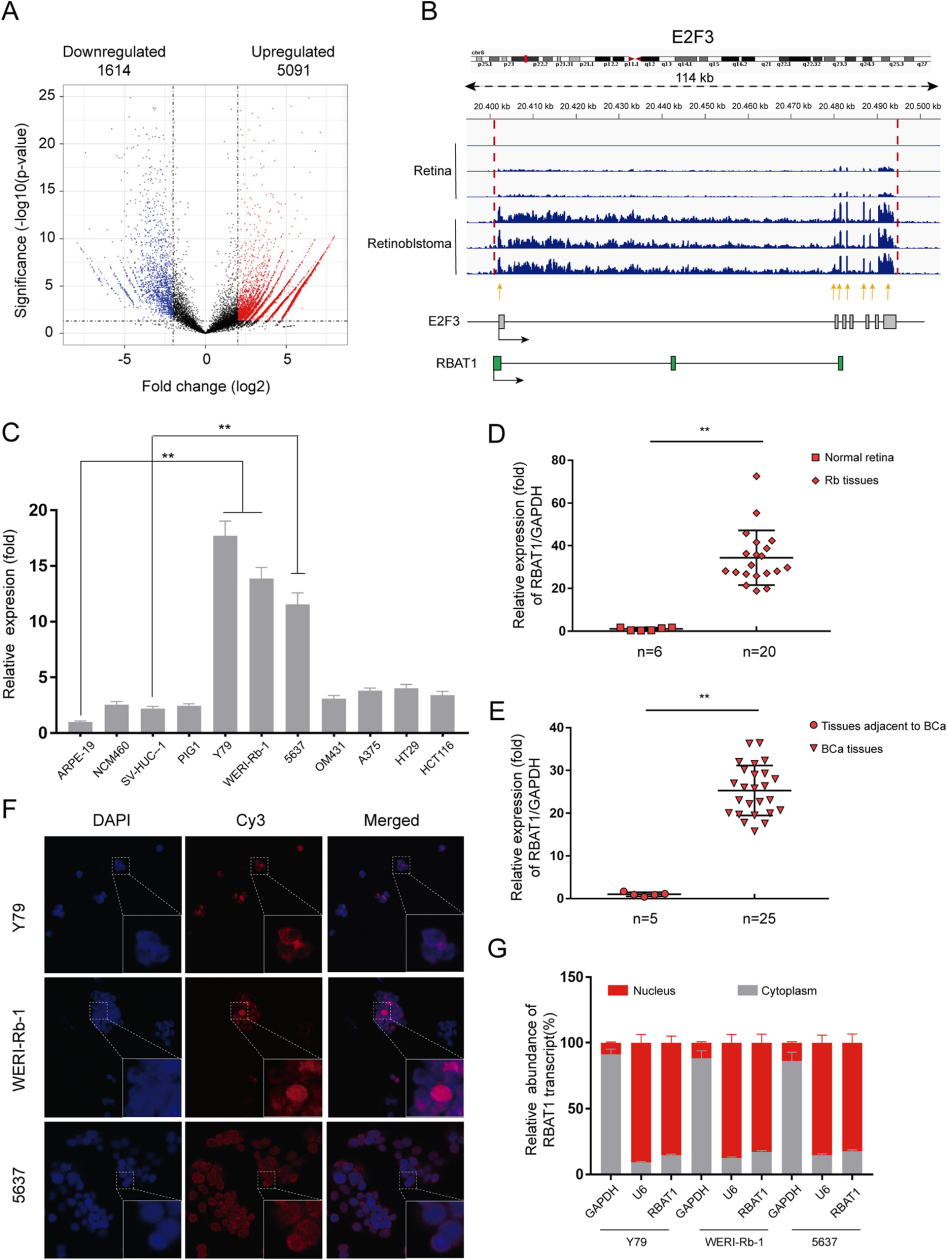
LncRNA RBAT1 is highly expressed in Retinoblastoma and Bladder Cancer cells. **a** Volcano plots of differentially expressed lncRNAs. The X-axis represents log fold changes. The Y-axis represents log *p* values. The blue points denote the significantly downregulated lncRNAs, and the red points denote the significantly upregulated lncRNAs. **b** RNA-sequence analysis was performed to evaluate the transcriptome in three retinoblastoma samples and three adjacent retina samples. Schematic annotation of the E2F3 genomic locus on Chr6p22.3; the gray rectangles represent the exons of E2F3, and the green rectangles represent RBAT1. **c** Real-time PCR analysis of RBAT1 expression in different tumor cell lines. *p < 0.05 and **p < 0.01. **d** and **e** RBAT1 expression levels in Rb tissues (**d**) and BCa tissues (e). RBAT1 was highly expressed in Rb and BCa tissues compared with normal tissues. **P < 0.01. **f** RNA FISH analysis shows that RBAT1 was located predominantly in the nucleus of Rb and BCa cell lines. **g** Cell nuclear/cytoplasmic fraction analysis and real-time PCR confirmed that RBAT1 was expressed mainly in the nucleus; GAPDH and U6 RNA served as positive controls for the cytoplasmic and nuclear fractions, respectively

Genome-wide RNA-Seq data (GEO, https://www.ncbi.nlm.nih.gov/geo/, GEO accession number: GSE111168) were used to identify lncRNAs that are aberrantly expressed in Rb tissues compared with adjacent normal tissues (*n* = 3). We found 5091 non-coding transcripts that were upregulated and 1614 non-coding transcripts that were downregulated as shown in Fig. 1a. Since 918 coding genes were upregulated and 760 coding genes were downregulated (Supplementary Fig. 1A), we concentrated on abnormally expressed lncRNAs, which share overlap regions with coding genes related to oncogenesis. We found 10 differential expressed genes (|log2(Fold_change)| > 1.5, *p* < 0.05) which were previously reported to be the driver/suppressor genes of retinoblastoma, and 14 differentials expressed lncRNAs (|log2FC| > 1.5, p < 0.05) were then identified which shared overlap region with these coding genes. Among these lncRNAs, we focused on the prominent one with highest expression of log2FC = 4.898, termed as ENST00000433182 (Supplementary Table 4). We further determined full-length ENST00000433182 transcripts using 5′ and 3′ RACE assay (Supplementary Fig. 1B). As shown in Supplementary Fig. 1B, only one transcript variant of RBAT1 was present in Rb tumor cell lines; this variant had a total length of 582 bp (Supplementary Fig. S2A) and lacking coding potential (Supplementary Fig. 2B and 2C), thereby we named it as retinoblastoma associated transcript-1 (RBAT1).

lncRNA RBAT1 is one of the most highly expressed lncRNAs in retinoblastoma (29.83-fold higher in Rb tissues than that in adjacent tissues), and RBAT1 resides on chr6p22.3, the promoter region of E2F3 gene (Fig. 1b). Previous study has indicated that E2F3 was highly expressed and contributed to tumor progression in a variety of tumors, such as colorectal [19, 20], melanoma [21–25] and bladder cancer [26–29]. We then examined RBAT1 expression in these tumor cell lines. The expression levels of RBAT1 in retinoblastoma and bladder cancer cell lines were much higher than that in other tumor cell lines (melanoma and colorectal cancer) (Fig. 1c). In addition, compared to that in normal tissues, RBAT1 was significantly upregulated in Rb and BCa tissues (Fig. 1d and e; Supplementary Tables 1 and 2). Furthermore, RBAT1 was localized mainly in the nucleus of tumor cell lines according to RNA fluorescence in situ hybridization (RNA FISH) (Fig. 1f) and cellular fractionation assays (Fig. 1g and Supplementary Fig. 2D). These findings suggested that lncRNA RBAT1, as a novel isoform of ENST00000433182, was highly expressed in the nucleus of Rb and BCa.

### Suppression of RBAT1 inhibits tumor progression in vitro and in vivo

**Fig. 2:**
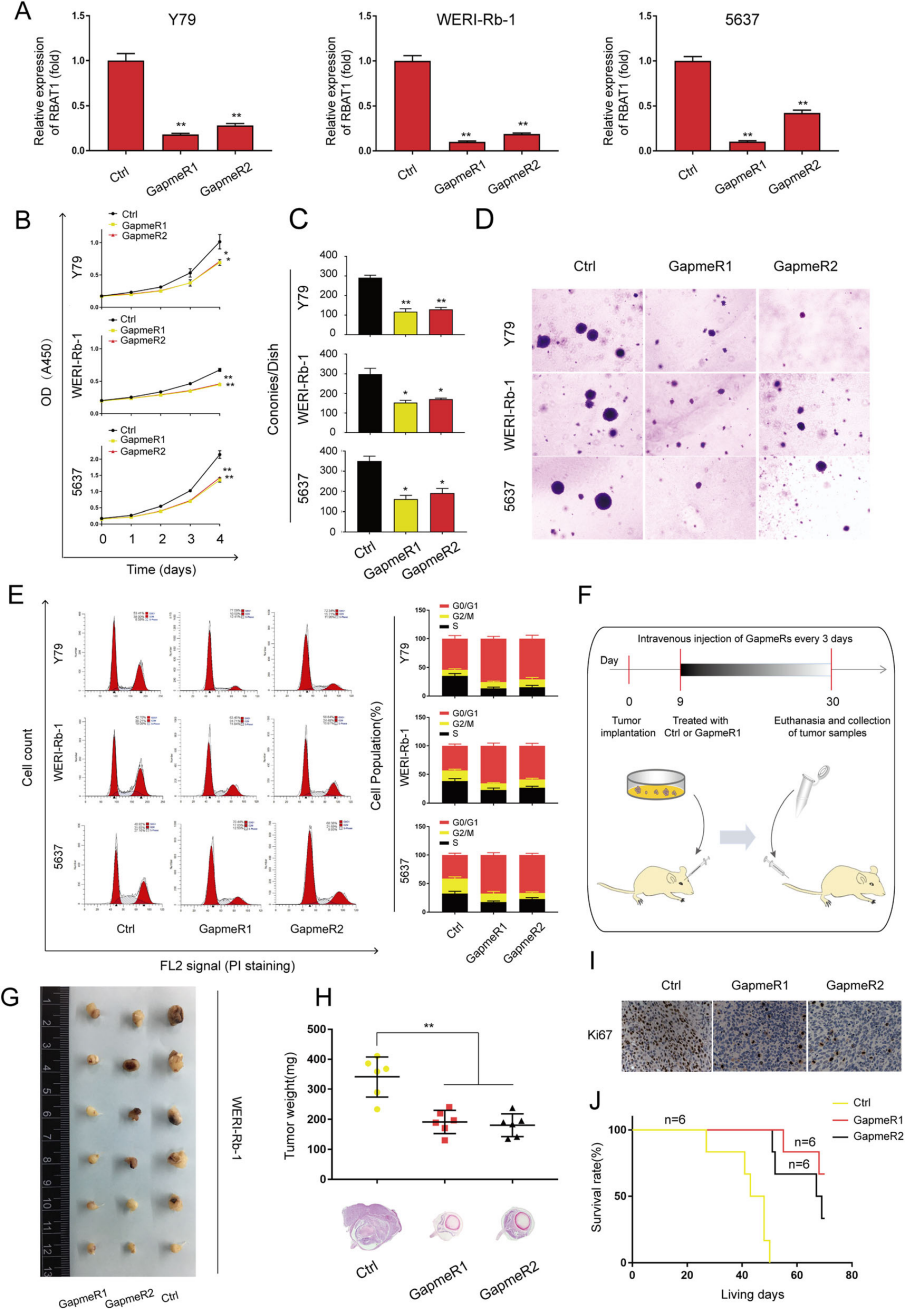
RBAT1 suppression inhibits tumor progression in vitro and in vivo. **a** RBAT1 was silenced in Rb and BCa cell lines by two independent GapmeRs (GapmeR1 and GapmeR2). Ctrl: negative control GapmeR. **b** A CCK8 assay was performed to assess cell proliferation in the control (Ctrl) and RBAT1 knockdown (GapmeR1 and GapmeR2) tumor cell lines. The results are shown as the mean ± SD in three independent experiments. *p < 0.05 and **p < 0.01. **c** and **d** A soft agar colony assay was performed to assess the tumor formation ability of RBAT1-silenced tumor cell lines (Y79, WERI-Rb-1 and 5637). Colony numbers were determined from three independent soft agar plates, the results are shown as the mean ± SD, *p < 0.05 and **p < 0.01. **e** Cell cycle analysis by flow cytometry was performed to determine the percentage of cells in different cell cycle phases. The percentage of cells in G0/G1 phase increased after RBAT1 knockdown in Y79, WERI-Rb-1 and 5637. X-axis represents FL2 channel-captured PI staining signals, and Y-axis represents cell counts. All histograms show the percentage (%) of cell populations from three independent experiments, the results are shown as mean ± SD. **f** Schematic of the orthotopic xenograft experiment. WERI-Rb-1 were injected into subretinal spaces of nude mice, which were then subjected to intravenous treatment with an RBAT1-targeting GapmeR (GapmeR1 and GapmeR2) or scrambled GapmeR (Ctrl) twice per week. **g** Representative image of tumor bearing eyeballs removed from the mice at the 30th day. **h** Top: Weights of tumors treated with GapmeR1 (*n* = 6), GapmeR2 (n = 6) and Ctrl (n = 6). Bottom: H&E staining to evaluate tumor formation. **p < 0.01. **i** Ki-67 staining of WERI-Rb-1 tumor tissues after intravenous treatment with GapmeR1, GapmeR2 and Ctrl. **j** Survival analysis of mice with orthotopic xenografts (WERI-Rb-1) treated with intravenous injections of GapmeR1, GapmeR2 and Ctrl twice per week; n = 6

To determine the role of RBAT1 in tumor progression, we silenced RBAT1 in tumor cell lines using LNA-modified antisense oligonucleotides (GapmeRs). These GapmeRs induced the RNase-H-mediated degradation of the target, significantly reducing RBAT1 expression in tumor cell lines (Fig. 2a). The control group and RBAT1-silenced group were designated Ctrl and GapmeR1/2, respectively. Notably, GapmeR1 achieved a higher knockdown efficiency. CCK8 assays showed that cell proliferation was significantly inhibited in RBAT1-silenced tumor cell lines (Fig. 2b). Consistently, the colony formation of tumor cell lines was decreased after silencing RBAT1 (Fig. 2c). We also observed that the RBAT1-silenced cell lines formed smaller colonies (Fig. 2d). Comparable results were observed in the colony formation assays (Supplementary Fig. 3A), however, it did not influence the migration of tumor cell lines (Supplementary Fig. 3B). Notably, after removing GapmeRs at day 3, sustained tumor inhibition was observed after RBAT1 depletion (Supplementary Fig. 3C). Moreover, we performed flow cytometry assays to determine whether RBAT1 was involved in cell cycle regulation and found that RBAT1 silencing induced G0/G1 cell cycle arrest in tumor cell lines (Fig. 2e).

To further investigate the role of RBAT1 in vivo, we injected WERI-Rb-1 Rb cells into subretinal spaces of nude mice to establish orthotopic xenograft models. Since GapmeR-mediated silencing has been reported to be efficacious both in vitro and in vivo [30], we thus performed intravenous treatment with RBAT1-targeting GapmeR1/2 (Fig. 2f). Compared with Ctrl group, RBAT1-silenced group had significantly inhibited tumor growth and reduced tumor volumes and weights (Fig. 2g and h). Moreover, immunohistochemistry staining showed that the tumors derived from RBAT1-silenced group exhibited lower expression of proliferation marker Ki67 than those from Ctrl group (Fig. 2i). RBAT1 expression in tumors of GapmeR1/2 groups were also decreased (Supplementary Fig. 3D). Additionally, we observed a significantly increased survival rate after silencing RBAT1 via GapmeR1/2 (Fig. 2j). Together, these data indicate that lncRNA RBAT1 plays an oncogenic role and that RBAT1 targeting might be a therapeutic approach for treating Rb patients.

### Silencing RBAT1 inhibits E2F3 signaling pathway

**Fig. 3:**
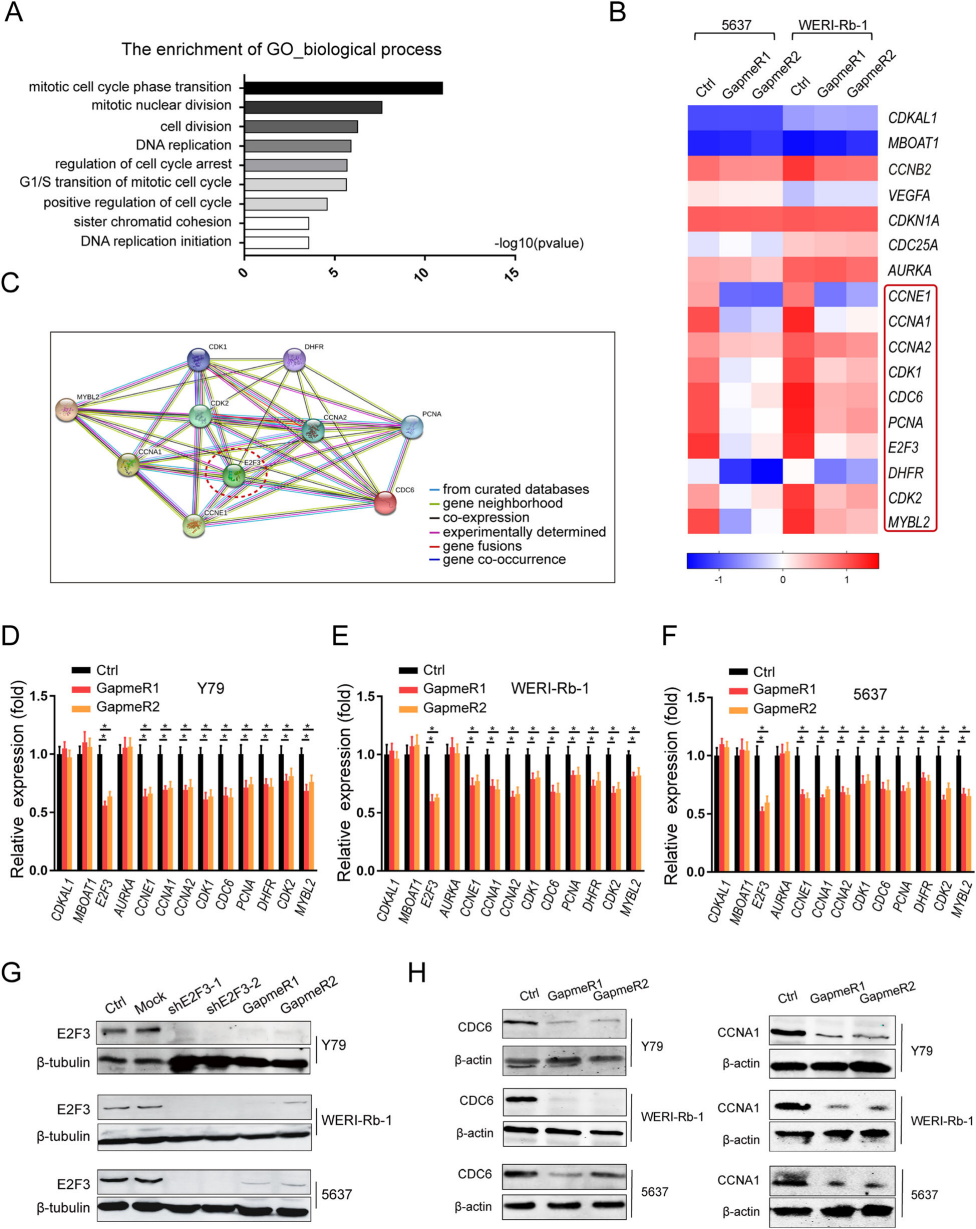
RBAT1 activates E2F3 signaling. **a** Gene ontology (GO) analysis was conducted to identify enriched biological processes in WERI-Rb-1. **b** A heat map shows the mRNA expression levels of several genes in RBAT1-silenced or Ctrl-treated WERI-Rb-1 cells and 5637 cells. CDKAL1 and MBOAT1 are the genes near E2F3, and the genes in red box are targets of E2F3. **c** An interaction analysis of the altered target genes in E2F3 signaling. **d**-**f** E2F3 and some of its downstream genes in the microarray were verified in Y79, WERI-Rb-1 and 5637 cells by real-time PCR. The results are shown as mean ± SD in three independent experiments. *p < 0.05. **g** shRNA knockdown efficacies were validated by western blot, and E2F3 protein levels were decreased after RBAT1 silencing by GapmeRs. **h** CDC6 and CCNA1 were analyzed by western blotting

To fully analyze the effect of RBAT1 on gene expression, we conducted transcriptome microarray analysis in Rb and BCa cell lines with or without RBAT1 silencing. RBAT1 knockdown resulted in differential expression of 1034 genes in BCa cell lines and 2262 genes in Rb cell lines (Supplementary Fig. 4A and 4B). Gene ontology (GO) analysis indicated that RBAT1 silencing resulted in significant inhibition of cell division, DNA replication, and cell cycle regulation (Fig. 3a, Supplementary Table 6). Notably, RBAT1 knockdown significantly reduced some genes that are involved in cell cycle and cell proliferation, including nearby protein-coding gene E2F3. In contrast, there were no significant changes in other neighboring genes, such as CDKAL1, MBOAT1 and other cell cycle regulators (Fig. 3b). To further confirm the target gene of RBAT1, we performed a gene interaction analysis of the altered genes. We observed that E2F3 played a key role in this gene regulatory network (Fig. 3c, Supplementary Fig. 4C and Supplementary Table 5).

We then determined the expression of the E2F3 signaling pathway after RBAT1 silencing in Y79, WERI-Rb-1 and 5637 cell lines. After RBAT1 inference, the E2F3 and its downstream targets were significantly downregulated at both mRNA (Fig. 3d-f) and protein (Fig. 3g and h) levels. These results indicated that E2F3 signaling is regulated by RBAT1.

### E2F3 accelerates tumor formation in vitro and in vivo

**Fig. 4:**
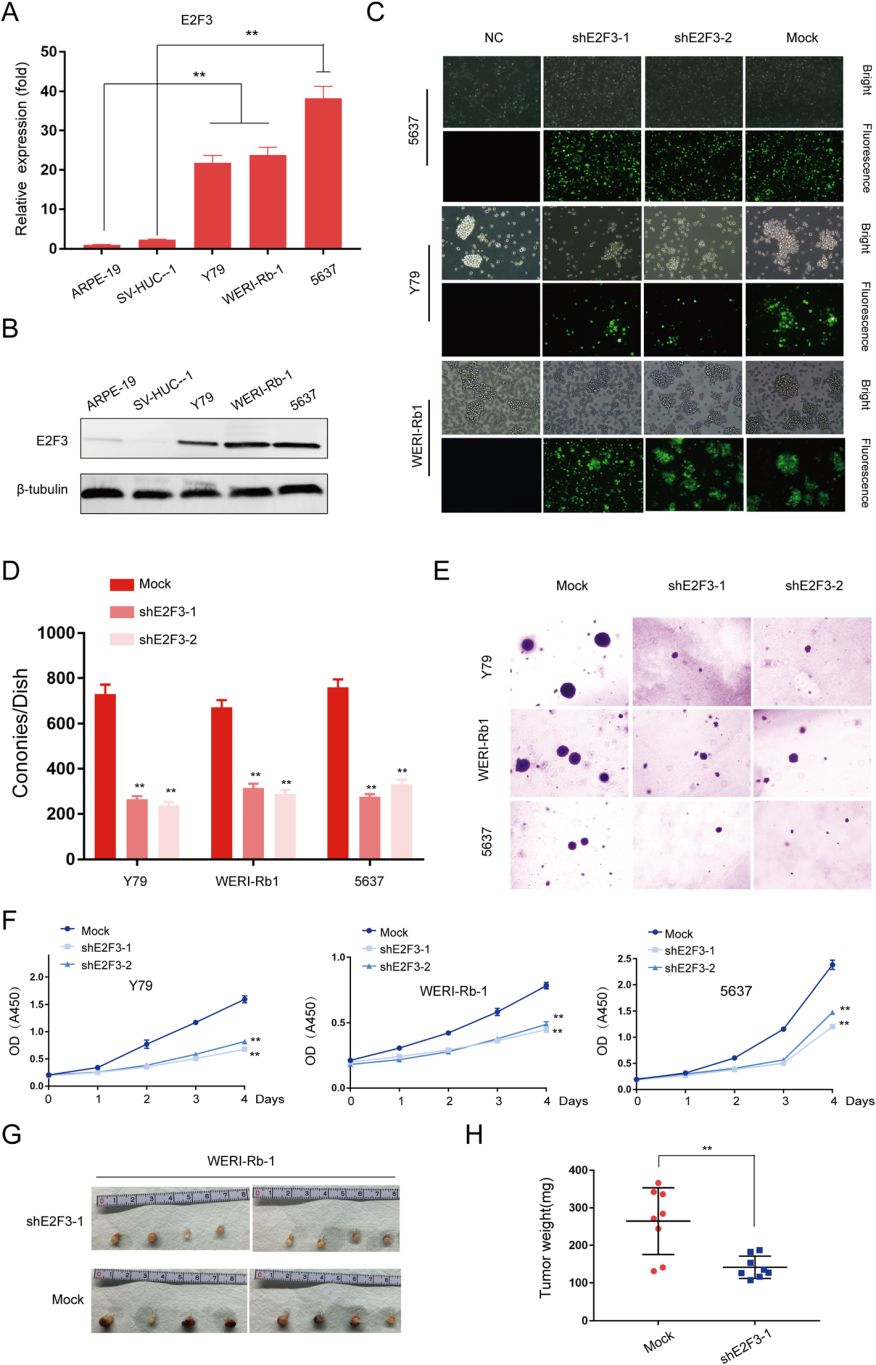
Suppression of E2F3 inhibits tumor progression in vitro and in vivo. **a** and **b** Real-time PCR (**a**) and western blot (**b**) showed higher expression levels of E2F3 in tumor cell lines compared with normal cell lines. **c** E2F3 knockdown by two independent shRNAs. EGFP was used to track the shRNA vectors (Mock, shE2F3–1, shE2F3–2) in Y79, WERI-Rb-1 and 5637 cell lines. **d** and **e** A soft agar colony assay was performed to assess tumor formation ability of E2F3-silenced tumor cell lines (Y79, WERI-Rb-1 and 5637). **f** CCK8 assay was performed to assess cell proliferation of E2F3-silenced tumor cell lines (Y79, WERI-Rb-1 and 5637). **g-h** General photograph (**g**) and the weight (**h**) of orthotopic xenograft at the 35th day after injection of WERI-Rb-1 into the subretinal space with or without E2F3 knockdown; *n* = 8. The results were shown as Mean ± SD, **p < 0.01

**Fig. 5:**
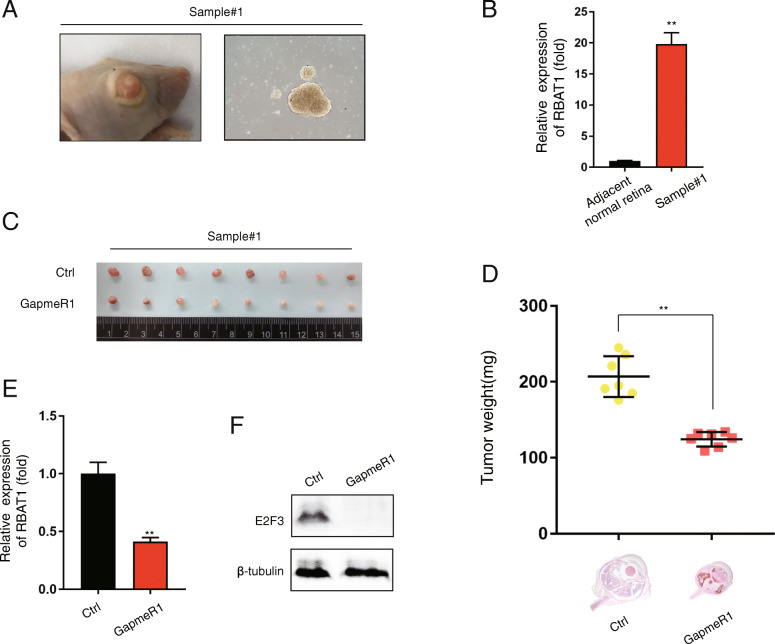
The therapeutic potential of RBAT1 targeted GapmeRs in primary Rb cells. **a** A patient-derived xenograft (PDX) model was established using Rb primary cells derived from patients. **b** Real-time PCR showed that the expression of RBAT1 is upregulated in Rb primary cells compared with normal tissues. **c-d** General photograph (**c**) of tumor bearing eyeballs removed from the mice at the 35th day. **d** Top: the weight of tumors treated by intravenous injection with RBAT1 targeted GapmeR (GapmeR1; n = 8), and scrambled GapmeR (Ctrl; n = 8). Bottom: H&E staining to evaluate tumor formation. **e-f** Real-time PCR (**e**) and western blot (**f**) analysis showed that the expression level of RBAT1 in orthotopic xenograft after intravenous treatment of lncRBAT1 targeted GapmeRs. The results were shown as Mean ± SD, **p < 0.01

To further explore the functional role of E2F3 in Rb and BCa cell lines, we first tested its expression. Here, we found that E2F3 expression is significantly higher in Rb and BCa cell lines than that in normal cell lines (Fig. 4a and b). As expected, the colony formation of tumor cell lines was decreased after E2F3 silencing (Fig. 4c, d). We also observed that the E2F3-silenced cell lines formed smaller colonies (Fig. 4e). CCK8 assays demonstrated inhibited proliferation after interfering E2F3 (Fig. 4f). To further evaluate the effects of E2F3 on Rb cell tumorigenesis in vivo, we performed orthotopic xenograft after E2F3 knockdown. Consistently, E2F3-silencing significantly reduced the tumor volumes and weights (Fig. 4g and h). Similarly, in a patient-derived xenograft (PDX) Rb model (Fig. 5a and b), RBAT1 inhibition had therapeutic effects (Fig. 5c and d) with decreased expression of E2F3 (Fig. 5e and f). Taken together, these data suggested that RBAT1 sustains tumor cell oncogenicity by activating E2F3 signaling.

Subsequently, we overexpressed RBAT1 and E2F3 in ARPE-19 and SV-HUC-1 cell lines (Supplementary Fig. 5A and B). We found that RBAT1 overexpression remarkably increased E2F3 expression in ARPE-19 and SV-HUC-1 cell lines (Supplementary Fig. 5C). We observed that the number of cell colonies was significantly increased in RBAT1-overexpressing and E2F3-overexpressed cell lines (Supplementary Fig. 5D and E). Additionally, cell proliferation was promoted in both RBAT1-overexpressing and E2F3-overexpressing cell lines (Supplementary Fig. 5F).

### RBAT1 interacts with HNRNPL and the E2F3 promoter region

**Fig. 6:**
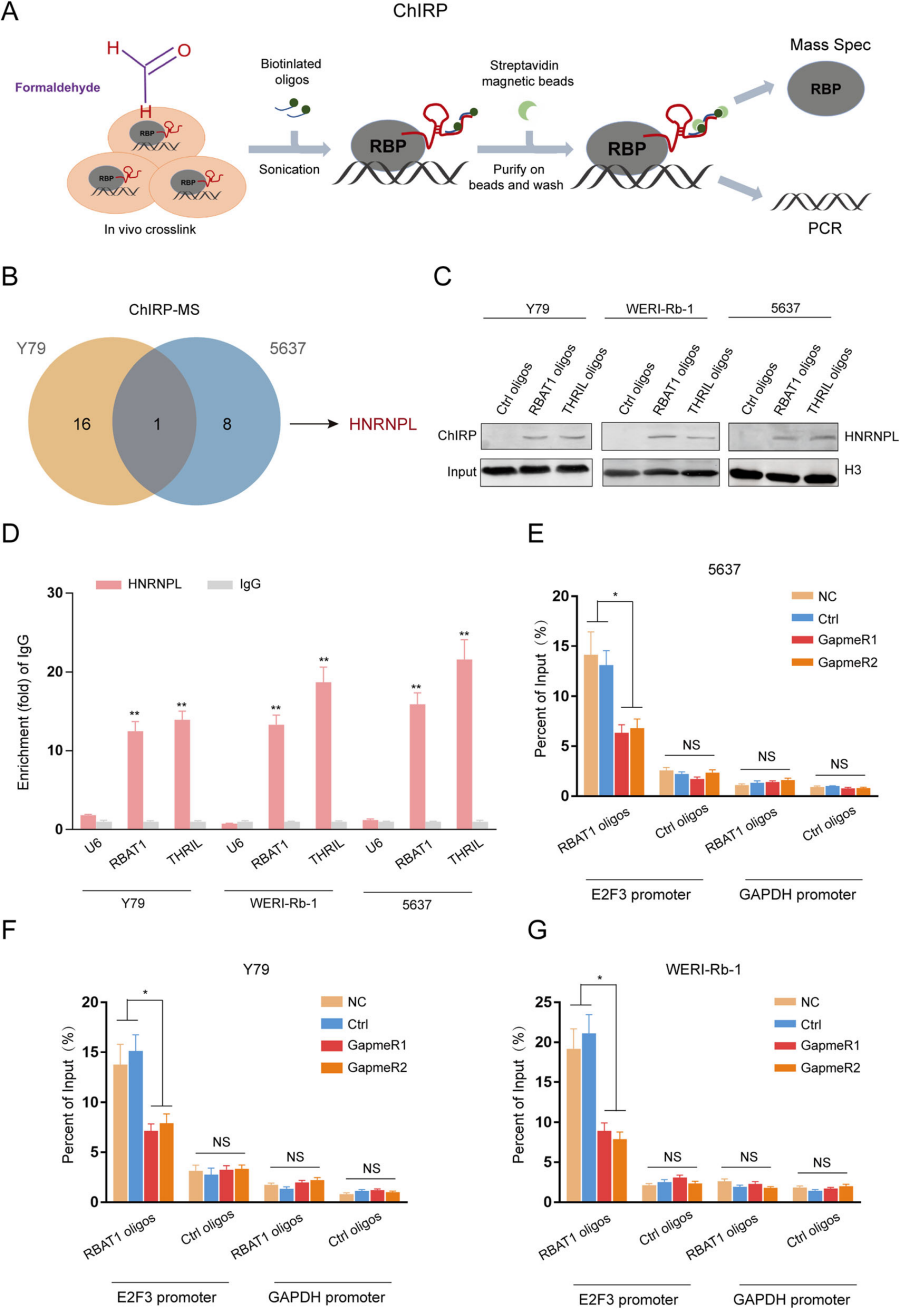
RBAT1 interacts with HNRNPL and E2F3 promoter region. **a** Technical description of the ChIRP method. **b** ChIRP-MS revealed that sixteen proteins bound to RBAT1 in Y79, and eight proteins bound to RBAT1 in 5637. HNRNPL was the only overlapping protein in Rb and BCa cell lines. **c** The ChIRP-MS results were verified by western blotting. RBAT1 oligos indicate the biotinylated antisense oligonucleotides against RBAT1. Ctrl oligos indicate the scrambled oligonucleotides, and THRIL oligos were selected as a positive control. **d** The interaction of RBAT1 with HNRNPL was verified by RNA immunoprecipitation (RIP) assays. The results are shown as the mean ± SD in three independent experiments. **p < 0.01. A validated HNRNPL interacting LncRNA THRIL served as a positive control. IgG antibody and U6 RNA served as negative controls. **e**-**g** ChIRP-PCR revealed that RBAT1 bound specifically to the E2F3 promoter. The GAPDH promoter region was selected as the negative control. NC: untreated cell lines; and Ctrl: cell lines transfected with GapmeR control. The results are shown as the mean ± SD in three independent experiments. NS means no significant difference, ** p < 0.01

LncRNAs are reported to exert their functions through interacting with RNA binding proteins (RBPs) that regulate gene expression by various mechanisms [31]. To explore the molecular mechanism underlying RBAT1-mediated E2F3 regulation, we performed ChIRP assays in Rb and BCa cell lines with biotin-labeled oligos to search for potential RBAT1-associated proteins (Fig. 6a). Seventeen ChIRP-purified proteins in Rb cell lines and nine proteins in BCa were identified by mass spectrometry (Supplementary Fig. 6; Supplementary Tables 7, 8). Importantly, only HNRNPL was found to bind specifically to RBAT1 in both protein subsets (Fig. 6b), and these results were confirmed by western blot analysis in three tumor cell lines (Fig. 6c). The interaction of RBAT1 with HNRNPL was further validated by RIP (Fig. 6d). Moreover, RBAT1 depletion did not alter HNRNPL expression levels (Supplementary Fig. 7A), suggesting that RBAT1 was not involved in the post-translational regulation of HNRNPL. Notably, using the BLAT (http://genome.ucsc.edu/), we found the sequence of lncRNA RBAT1 could not only align to E2F3 promoter (chr6:20401729–20,481,937, 100% similarity), but also 5 kb upstream from TSS of SLFN12L (chr17:35490212–35,490,325, 94.8% similarity) and a intronic region of DNAH7 (chr2:195995428–195,995,503, 87.1% similarity), which would be potential binding sites for lncRNA RBAT1 (Supplementary Fig. 8). Given that the first exon of RBAT1 was in the E2F3 promoter and that a 209 bp complete complementary sequence is found in this region, the E2F3 promoter was chosen as the candidate site for detection. The GAPDH promoter served as a negative control. We analyzed a 2 kb locus upstream from the transcription start site (TSS) of the E2F3 gene to confirm the promoter region (Supplementary Fig. 9A). Through luciferase reporter assays, we identified a − 500 to − 1000 bp segment upstream from the TSS as the promoter region of E2F3 with high transcriptional activity (Supplementary Fig. 9B). Specifically, ChIRP-PCR showed that RBAT1 was enriched in the E2F3 promoter region in three tumor cell lines, whereas a weak RBAT1 enrichment was observed at the E2F3 promoter after RBAT1 suppression (Fig. 6e-g); suggesting that the promoter region of E2F3 was the binding site for lncRNA RBAT1. These data indicate that RBAT1 binds directly to HNRNPL and the E2F3 promoter region with high affinity.

### RBAT1 recruits HNRNPL to activate E2F3 expression

**Fig. 7:**
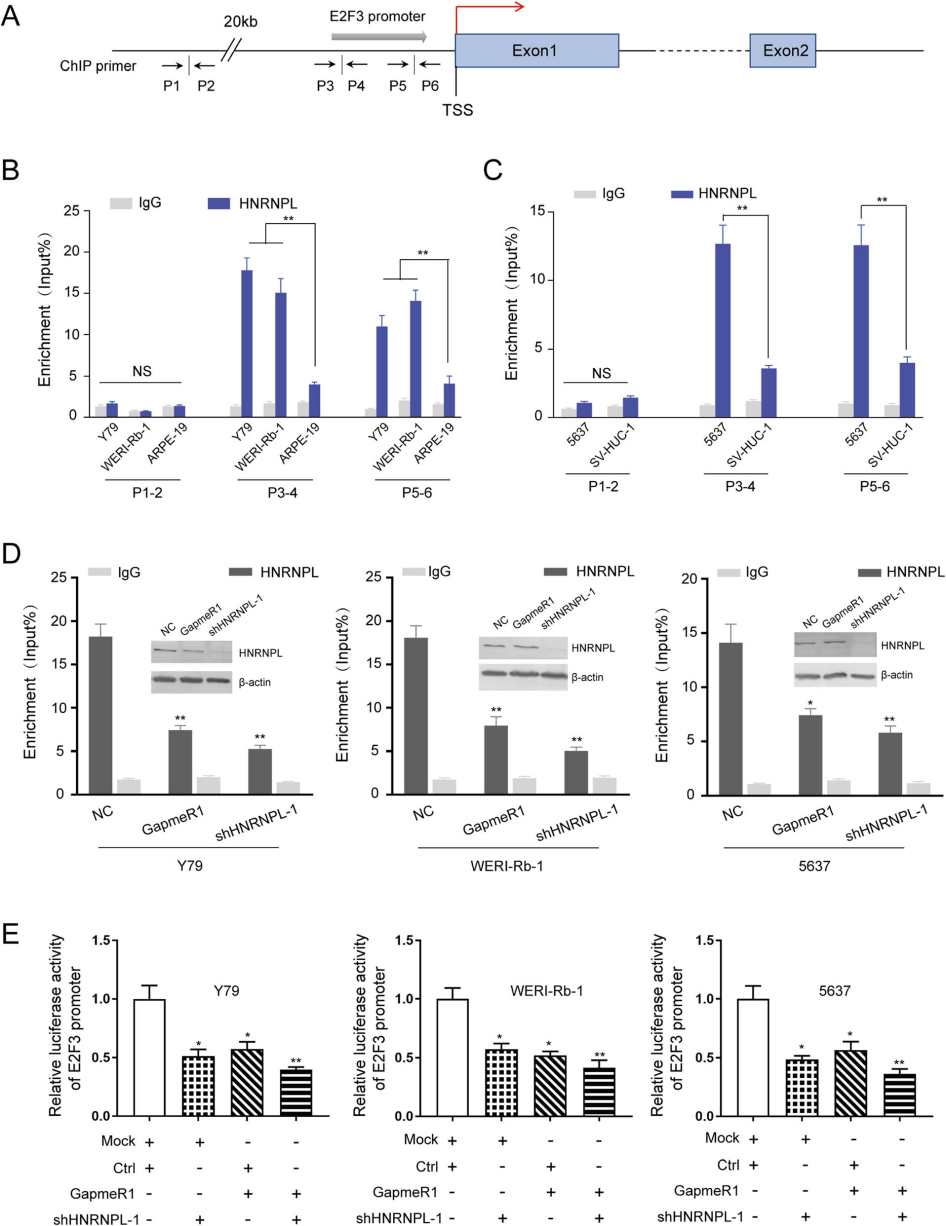
RBAT1 recruits HNRNPL to activate E2F3 expression. **a** Schematic diagram of ChIP assay sites in E2F3 promoter region. P1-P5: primer names; gray arrow: E2F3 promoter region (1000 bp upstream from TSS; P3-P4, P5-P6: different ChIP sites of E2F3 promoter); red arrow: transcriptional direction; blue box: exons of the E2F3 gene. **b**-**c** Real-time PCR showing the interaction of HNRNPL with E2F3 promoter in tumor cell lines (Y79, WERI-Rb-1 and 5637) compared with ARPE-19 and SV-HUC-1 from ChIP assay. IgG: negative control. The results are shown as the mean ± SD. NS means no significant difference, ** p < 0.01. **d** ChIP analysis showed that the depletion of either RBAT1 or HNRNPL decreases the enrichment of HNRNPL at E2F3 promoter. The knockdown efficiency of shHNRNPL-1 was verified by western blotting, and RBAT1 knockdown did not influence the expression level of HNRNPL. IgG: negative control. **e** Verified E2F3 promoter sequences (− 500 to − 1000 bp) were constructed into a pGL3 vector and subjected to luciferase reporter assays in either RBAT1- or HNRNPL-silenced tumor cell lines (Y79, WERI-Rb-1 and 5637). Mock: negative control group transfected with empty pGL3 vector; Ctrl: negative control group transfected with scrambled GapmeR. The results are shown as the mean ± SD, *p < 0.05 and **p < 0.01

Given that HNRNPL could regulate gene transcription by forming a lncRNA-HNRNPL complex and bind to promoter of TNFα [32], we proposed a hypothesis that RBAT1/HNRNPL may activates E2F3 expression via binding to its promoter region. We next performed ChIP assays and found that HNRNPL bound to E2F3 promoter in tumor cell lines (10 ~ 20-fold changes), while there are merely 2 ~ 4-fold changes in ARPE-19 and SV-HUC-1 (Fig. 7a-c). Furthermore, independently knocking down HNRNPL (Supplementary Fig. 7B) or RBAT1 reduced HNRNPL binding to E2F3 promoter (Fig. 7d), indicating that RBAT1 depletion reduced the binding capacity of HNRNPL to E2F3 promoter. To determine whether RBAT1 could activate E2F3 by recruiting HNRNPL to its promoter, we evaluated the status of E2F3 promoter transcriptional activity in tumor cell lines with different RBAT1 and HNRNPL expression levels using luciferase assays. We observed that E2F3 promoter activation was suppressed in RBAT1 or HNRNPL knockdown tumor cell lines (Fig. 7e). Also, we examined the DNA copy number variations (CNV) in tumor cell lines. We observed an increased E2F3 copy number in Rb and BCa cell lines. However, there was no obvious changes in the copy number of E2F3 gene after RBAT1 silenced (Supplementary Fig. 10 and 11). Furthermore, we have also tested HT1376, a bladder cancer cell line without E2F3 locus amplification (Supplementary Fig. 12A). We found that RBAT1 was also highly expressed in HT-1376 (Supplementary Fig. 12B) which indicates the RBAT1 was not upregulated by copy number amplification of E2F3 locus. In addition, after knockdown RBAT1 (Supplementary Fig. 12C), the expression of E2F3 was largely reduced (Supplementary Fig. 12D) and the tumor proliferation was also suppressed (Supplementary Fig. 12E). Our results indicating that RBAT1 was not upregulated by copy number amplification of E2F3 locus, could also epigenetically regulate E2F3 expression. To sum up, our data showed that RBAT1 recruits HNRNPL to E2F3 promoter and activates its expression.

### The E2F3/RBAT1 cluster harbors clinical significance in tumors

**Fig. 8:**
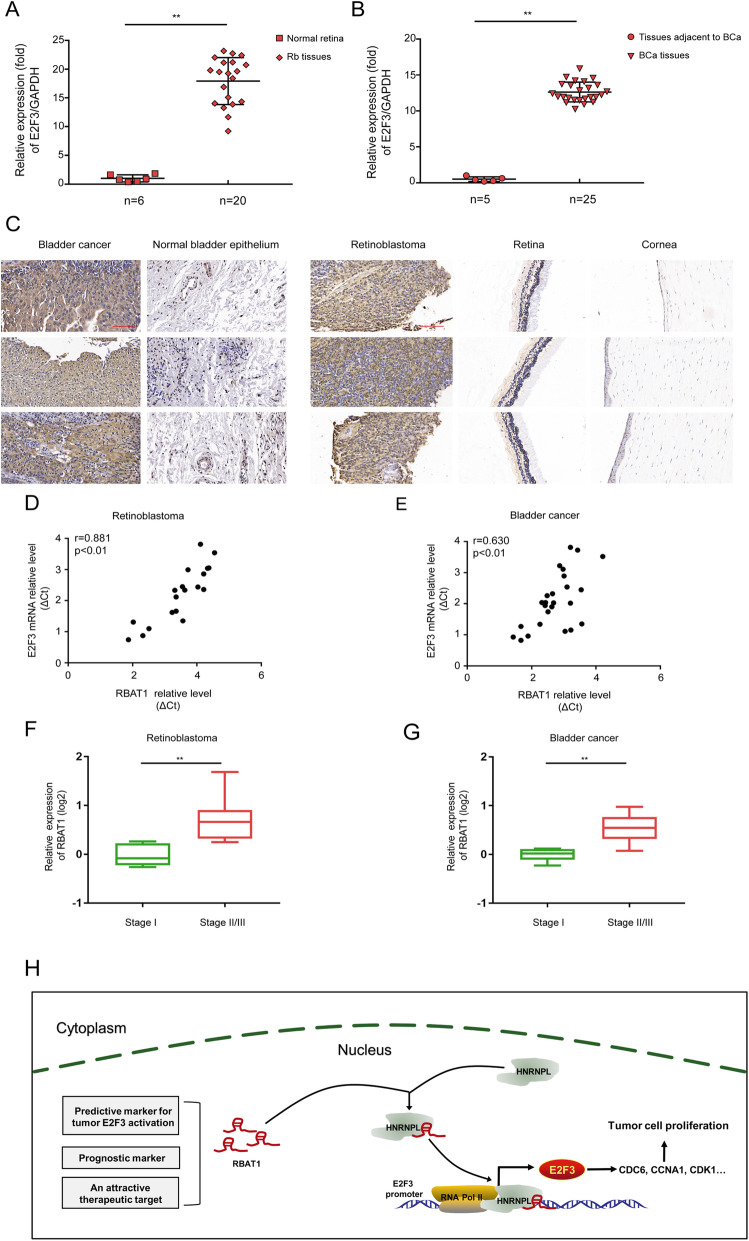
The clinical significance of RBAT1 and E2F3 in tumors. **a** and **b** E2F3 expression levels in Rb tissues (**a**) and BCa tissues (**b**). E2F3 was highly expressed in Rb and BCa tissues compared with normal tissues; **P < 0.01. **c** Immunohistochemistry staining of tumor sections from patients showed that E2F3 expression was higher in tumor tissues than that in normal tissues; scale bar: 100 μm. (**d** and **e**) Expression levels of RBAT1 and E2F3 were detected in 20 Rb samples (**d**) and 25 BCa samples (**e**) subjected to correlation analysis. The data were normalized to GAPDH expression and are shown as the ΔCT. **f** and **g** Box plot representing RBAT1 expression in Rb (**f**) and BCa (**g**) specimens at stage I (Rb: *n* = 5; BCa: *n* = 6) and stage II/III/IV (Rb: *n* = 15; BCa: *n* = 19). Patients were classified according to the International Retinoblastoma Staging System (IRSS). ** p < 0.01. **h** Schematic model of the lncRNA-RBAT/HNRNPL complex in E2F3 promoter induced oncogene activation and tumorigenesis. Grey circle: HNRNPL protein; and red circle: E2F3 protein

We further explored the potential clinical significance of the E2F3/RBAT1 cluster. Real-time PCR showed that the mRNA expression levels of RBAT1 and E2F3 were higher in tumors than that in adjacent tissues in both Rb and BCa (Fig. 8a and b). Similarly, higher E2F3 expression in Rb and BCa samples was further confirmed by immunohistochemistry staining (Fig. 8c). Notably, RBAT1 expression was positively correlated with E2F3 expression in both Rb (Fig. 8d) and BCa (Fig. 8e) samples. We then assessed the clinical relevance of E2F3 and RBAT1. Importantly, high expression levels of RBAT1 and E2F3 correlated with advanced clinical stages of Rb and BCa (Fig. 8f and g; Supplementary Fig. 13A). Moreover, a Kaplan-Meier survival analysis indicated that higher survival probability was associated with lower expression of E2F3 (Supplementary Fig. 13B and C). These data further highlight the clinical importance of RBAT1 and E2F3 in Rb and BCa.

## Discussion

Recently, numerous non-coding transcripts have been discovered in diverse types of cancers through high-throughput RNA sequencing technologies. LncRNAs have been reported to play an leading role in cancer initiation and progression [33, 34]. LncRNAs exert their functions via various mechanisms, including modulating gene expression, modifying chromatin, pairing with other RNAs, and acting as scaffolding for nuclear or cytoplasmic functional complexes [35]. Notably, increasing evidence has shown that lncRNAs may act as epigenetic modifiers to regulate gene expression [36, 37]. Here, we reveal a novel lncRNA which accelerates tumorigenesis via recruiting HNRNPL to the promoter of an oncogene, E2F3, and activating its expression (Fig. 8h).

It should be emphasized that ENST00000433182 is 400 bp in length, according to the University of California, Santa Cruz (UCSC), and NCBI database. In our study, we present a novel 582 bp isoform of ENST00000433182 with three exons. More precisely, exon 1 lies in the promoter region of E2F3, while exon 2 and exon 3 locate in the intronic region of the E2F3 gene locus and we named it as RBAT1. Interestingly, we noted that a 209-nucleotide region in RBAT1 was completely complementary to the genomic sequence of the E2F3 promoter, which increased the accessibility for RBAT1 to recruit functional complexes to E2F3 promoter region. However, the role of RBAT1 has not been elucidated. Notably, we found the sequence of lncRNA RBAT1 could not only align to E2F3 promoter, but also 5 kb upstream from TSS of SLFN12L and an intronic region of DNAH7, which would become potential binding site of lncRNA RBAT1. Importantly, SLFN12L has been revealed to regulate intestinal epithelial differentiation and contribute to prostate cancer cell differentiation [38, 39]. In addition, DNAH7 was identified as novel susceptibility genes in testicular cancer [40]. Here, we have only validated the interaction between lncRNA RBAT1 and E2F3 promoter. The possibility of lncRNA RBAT1 guided trans-regulation, such as regulating SLFN12L and DNAH7, requires further exploration.

Since we identified that RBAT1 could cis-regulate E2F3, we next explored the molecular mechanism underlying RBAT1-induced E2F3 transcriptional activation. In our study, we identified that the lncRNA binding factor HNRNPL, a member of the RBP family, plays a key role in the transcriptional regulation of E2F3. Notably, HNPNPL has been reported previously to be involved in alternative splicing events and play a vital role in cellular processes [41]. Recent studies have focused mainly on the post-transcriptional functions of HNRNPL in tumorigenesis [9, 42, 43]. For example, HNRNPL could directly regulate RNA processing, including androgen receptor RNA alternative splicing and circular RNA formation in prostate cancer [42]. Although we cannot exclude that other genetic or epigenetic factors regulate E2F3 expression, this is the only study to indicate that E2F3 is regulated by HNRNPL. In conclusion, we found that RBAT1 and HNRNPL formed a complex at the E2F3 promoter and cis-activated its expression, thereby providing novel insight into this oncogene activation in both Rb and BCa.

It should also be noted that the E2F Transcription Factor 3 (E2F3) gene, a member of the E2F transcription factor family, regulates the cell cycle-dependent expression of genes that are essential for cellular proliferation [44, 45]. Although Rb and BCa are two distinct tumors with different genetic and epigenetic backgrounds, we found that they share the feature of E2F3 activation. We found E2F3 mRNA was decreased after silencing RBAT1 without CNV changes in this locus, which indicate that RBAT1 could regulate E2F3 expression in a CNV-independent manner and transcriptional activation may plays an important role in E2F3 overexpression.

Previous studies have revealed increased E2F3 expression in different types of tumors, such as lung, prostate, bladder and retinoblastoma [46–48]. In addition, epigenetic factors, such as miR-195 and miR-148a, could also target E2F3 mRNA, downregulate its expression and result in tumor growth inhibition [49, 50]. However, the relationship between lncRNA and E2F3 remains unclear. Here, we use two types of E2F3-activated tumors, retinoblastoma and bladder cancer, to demonstrate that RBAT1 upregulation contributed to the activation of E2F3 in these tumor cells. Since E2F3 activation is a common feature across diverse types of tumors, we propose that RBAT1-induced E2F3 activation might be a widespread novel mechanism in tumors. Of note, we observed a 50% decrease in E2F3 RNA but a more than 90% decrease in protein after silencing RBAT1. Previous study demonstrated that the changes of mRNA were not always paralleled to that of protein. For example, TCF7-targeted shRNA resulted in 50% decrease in TCF7 mRNA while more than 90% in protein [51].

Retinoblastoma develops in response to the biallelic loss of RB1 gene [52–54]. Typically, the wild-type RB protein could bind and deactivate E2F3 protein, while mutated RB protein failed to silence E2F3 protein and contributed to cell cycle dysfunction [55]. In conclusion, RB1 mutations lead to E2F3 deregulation at the post-translational level. However, the reason for E2F3 mRNA upregulation in retinoblastoma remains unclear. As control cell (ARPE19 and SV-HUC-1) were immortal cell lines without defined biologic relationship with corresponding tumor cell lines, the biologic comparison of E2F3 expression in tumors and control cell lines should be further determined. In clinical samples, we concluded that mRNA and protein of E2F3 is overexpressed in tumors. Here, we demonstrated that E2F3 overexpression may be mediated by RBAT1, which provides a novel lncRNA-mediated mechanism underlying Rb tumorigenesis.

GapmeRs are chimeric antisense oligonucleotides containing central blocks of deoxynucleotide monomers. GapmeRs are sufficiently long to induce RNase H cleavage [30, 56]. Due to their stability in biological fluids, GapmeR antisense oligonucleotides were designed to selectively cleave the targeted lncRNAs. It has been proven to be efficient in inhibiting lncRNAs [30]. For example, intravenous treatment with a lncRNA SAMMSON-targeting GapmeR significantly inhibited tumor growth in PDX models of melanoma [30]. In this study, we revealed that RBAT1-targeting GapmeRs decreased Rb formation in orthotopic xenograft retinoblastoma derived from both Rb cell lines and Rb primary cells. Given that the GapmeR antisense oligonucleotides are bioactive small molecules that could freely cross the blood-eye barrier, antisense drugs targeting RBAT1 may be a promising therapeutic approach for retinoblastoma.

## Conclusion

Retinoblastoma (Rb) is the most common primary intraocular malignancy in children. It has been proven to be associated with biallelic loss of RB1 gene, aberrant copy number variation and epigenetic dysregulations. Our study revealed retinoblastoma associated transcript-1 (RBAT1), one of the top upregulated lncRNAs in Rb, functions as an oncogene by accelerating tumor cell growth and formation. LncRNA-RBAT1 could recruit HNRNPL to E2F3 promoter, resulting in activating of E2F3 signaling pathway. Therapeutically, GapmeR-mediated RBAT1 silencing inhibited tumorigenesis in orthotopic xenograft retinoblastoma models derived from Rb cell lines and Rb primary cells. Targeting RBAT1/E2F3 provides a therapeutic strategy in the treatment of Rb.

## Supplementary information

**Additional file 1: Supplementary Table 1.** Clinicopathological features and demographics of retinoblastoma (Rb) patient cohorts. **Supplementary Table 2.** Clinicopathological features of Bladder Urothelial Carcinoma (BCa) patient cohorts. **Supplementary Table 3.** Primers, oligos, shRNAs, GapmeRs and probes used in the experiment. **Supplementary Table 4.** The list of lncRNAs that shared an overlap region with tumor related genes in retinoblastoma. **Supplementary Table 5.** Interactions between E2F3 and its targeting genes. **Supplementary Table 6.** Information of GO (Biological Process) Enrichment Analysis. **Supplementary Table 7.** ChIRP-MS identified lncRNA RBAT1 specifically binding proteins in Y79. **Supplementary Table 8.** ChIRP-MS identified lncRNA RBAT1 specifically binding proteins in 5637. **Fig. S1.** RBAT1 is a novel transcript in retinoblastoma (Rb) and bladder cancer (BCa). (A) Volcano plots of differentially expressed protein-coding genes. X axis represents log fold changes. Y axis represents log *p* values. Blue points denote significantly downregulated genes and red points denote significantly upregulated genes. (B) Agarose gel electrophoresis of PCR products generated by 3′ (left) and 5′ (right) RACE. (C) Schematic diagram of RACE assay. **Fig. S2.** RACE and coding potential analyses of RBAT1. (A) The full sequence of RBAT1. (B) Coding Potential Calculator (CPC) analysis suggested that RBAT1 is a noncoding RNA. GAPDH, CRTC1 and ACTB genes were used as protein-coding controls. XIST and HOTAIR genes were used as noncoding RNA controls. (C) RBAT1 was predicted by PhyloCSF to have no protein coding potential. The peaks showed the PhyloCSF score for each codon in each of 6 frames. Regions with a score less than 0 are predicted to be noncoding while Regions with a score greater than 0 are predicted to be coding. The protein coding gene ACTB and the noncoding RNA gene HOTAIR were used as controls. (D) Fractionation of tumor cell lines (Y79, WERI-Rb-1 and 5637) followed by RT-PCR. RBAT1 was mainly expressed in the nucleus. GAPDH and U6 RNA served as positive controls for the cytoplasmic and nuclear fractions, respectively. **Fig. S3.** Effect of RBAT1 on colony formation ability and migration ability of tumor cell lines. (A) A colony formation assay was performed to determine the colony formation ability of RBAT1-silenced tumor cell lines (Y79, WERI-Rb-1 and 5637). For colony formation assays, 500 cells were seeded in 6-well plates (Poly-L-lysine-coated 6-well plates for retinoblastoma cell lines). 7–14 days later, the colonies were washed with PBS, fixed and stained for 20 min with a 1% crystal violet solution. Images were captured by a scanner, and the percentage of cell occupancy was counted and analyzed by ImageJ software. (B) The migration and invasion abilities displayed no significant changes in RBAT1-silenced tumor cell lines (5637) compared with ctrl group. (C) A colony formation assay was performed to test sustained effect after removing GapmeRs at the 3rd day. (D) A real-time PCR was performed to determine the expression level of RBAT1 in tumors from GapmeR1/2 treated groups and Ctrl. The results are shown as the mean ± SD in three independent experiments. *p < 0.05 and **p < 0.01. **Fig. S4.** Gene expression patterns associated with cell proliferation in RBAT1-silenced Rb and BCa cell lines. (A) Gene expression profiles in 5637 cells and WERI-Rb-1 cells transfected with GapmeR1/2 or a ctrl GapmeR. (B) The interaction of genes in cell cycle pathway. The down-regulated genes in red semitransparent area were verified in our study. **Fig. S5.** The overexpression of RBAT1 and E2F3 in normal cell lines. (A) Realtime-PCR showed the expression levels of RBAT1 after transfecting pcDNA3.1- RBAT1 in ARPE-19 and SV-HUC-1. (B-C) Western blot was performed to test E2F3 expression after transfecting pcDNA3.1-RBAT1 or pcDNA3.1-E2F3 in ARPE-19 and SV-HUC-1, respectively. (D-E) Colony formation assays were performed to measure the colony formation ability of ARPE-19 and SV-HUC-1 with RBAT1 or E2F3 overexpression. (F) CCK8 assay was performed to assess cell proliferation of RBAT1 or E2F3 over-expressed normal cell lines (ARPE-19 and SV-HUC-1). The results were shown as Mean ± SD, *p < 0.05; **p < 0.01. **Fig. S6.** ChIRP-MS analysis of RBAT1-interacting proteins. The protein peptides isolated by ChIRP. U6 was selected as control, and scrambled oligos were selected as negative controls. **Fig. S7.** HNRNPL expression analysis. (A) Western blot analysis showed that RBAT1 depletion did not influence the expression level of HNRNPL. (B) HNRNPL was silenced in tumor cell lines (Y79, WERI-Rb-1 and 5637) by two independent shRNAs. **Fig. S8.** Alignment of RBAT1 sequences and human genome sequences. lncRNA RBAT1 could not only align to E2F3 promoter (chr6:20401729–20,481,937, 100% similarity, 1st panel), but also 5 kb upstream from TSS of SLFN12L (chr17:35490212–35,490,325, 94.8% similarity, 2nd panel) and an intronic region of DNAH7 (chr2:195995428–195,995,503, 87.1% similarity, 3rd panel). **Fig. S9.** Identification of the E2F3 promoter region. (A) Schematic of the luciferase assay of E2F3 promoter. 2000 bp upstream to E2F3 TSS was selected for detection. The full-length 5′ to 3′ sequence was used as positive control (lane a). The untreated group (NC) and empty vector group (Mock) served as negative control groups. Different segments of E2F3 promoter (lane b-e) were constructed into pGL3 vector and subjected to luciferase reporter assays in HEK-293 T cells. Through luciferase reporter assays, we identified a − 500 to − 1000 bp segment of TSS upstream region as the core promoter of E2F3 with transcriptional activity. **Fig. S10.** Copy number variation (CNV) analysis of Rb cell lines. (A and B) CNV microarray showed increased E2F3 gene copy number in Y79 and WERI-Rb-1 compared with ARPE-19. RBAT1 interference did not influence the CNV of retinoblastoma cell lines. The Affymetrix OncoScan CNV microarray was used to detect the CNV of cells. The arrays were scanned with a GeneChip Scanner 3000 7G System and the data were analyzed using Chromosome Analysis Suite (ChAS). **Fig. S11.** Copy number variation (CNV) analysis of BCa cell lines. (A and B) CNV microarray of bladder cancer cell line showed increased E2F3 gene copy number in 5637 compared with SV-HUC-1. RBAT1 silencing did not influence the CNV of bladder cancer cell lines. **Fig. S12.** HT1376, a bladder cancer cell line without E2F3 copy number amplification, represent highly-expressed RBAT1. (A) HT-1376, a bladder cancer cell line without E2F3 locus amplification. (B) Real-time PCR showed that RBAT1 was also highly expressed in HT-1376. (C) Real-time PCR showed that RBAT1 was knockdown by GapmeR1. (D and E) After RBAT1 knockdown the expression of E2F3 was largely reduced (D) and the tumor proliferation was also suppressed (E). The results were shown as Mean ± SD, **p < 0.01. **Fig. S13.** E2F3 associates with Rb and BCa clinical characteristics and predict disease prognosis. (A) Box plot represented E2F3 expression in Rb (left) and BCa (right) specimens at stage I (Rb: *n* = 5; BCa: *n* = 6) and at stage II/III/IV (Rb: *n* = 15; BCa: *n* = 19). *p < 0.05; **p < 0.01. (B) Kaplan-Meier survival analysis was performed in 224 cases of bladder cancer using R2 platform (https://hgserver1.amc.nl). (C)The normalized expression value of E2F3 in 224 BCa patients were automatically calculated in R2 platform.

## Data Availability

RNA-Seq of retinoblastoma samples have been uploaded to public databases (GEO, https://www.ncbi.nlm.nih.gov/geo/, GEO accession number: GSE111168). The microarray data after RBAT1 deletion has been uploaded in National Omics Data Encyclopedia (NODE) database (http://www.biosino.org/node/project, accession number: OEP000762).

## Abbreviations

RBAT1: retinoblastoma associated transcript-1
Rb: retinoblastoma
BCa: bladder cancer
ChIRP: chromatin isolation using RNA purification
PDX: patient-derived xenograft
E2F3: E2F transcription factor 3
HNRNPL: heterogeneous nuclear ribonucleoprotein L
PRC2: polycomb repressive complex 2
LncRNAs: long noncoding RNAs
CPC: coding potential calculator
RIP: RNA immunoprecipitation
ChIP: chromatin immunoprecipitation
FISH: fluorescence in situ hybridization
TSS: transcription start site
TNF-α: tumor necrosis factor α
CNV: copy number variations
shRNA: short hairpin RNA
STAT3: signal transducer and activator of transcription 3
RACE: rapid amplification of cDNA ends
TCEA1: transcription elongation factor A1
