# Supplementary material for: A novel LncRNA transcript, RBAT1, accelerates tumorigenesis through interacting with HNRNPL and cis-activating E2F3
Source: Mol Cancer. 2020 Jul 15;19:115. doi: 10.1186/s12943-020-01232-3 (PMC7362570; doi:10.1186/s12943-020-01232-3)
Supplement: Supplementary file 1 — Additional file 1: Supplementary Table 1. Clinicopathological features and demographics of retinoblastoma (Rb) patient cohorts. Supplementary Table 2. Clinicopathological features of Bladder Urothelial Carcinoma (BCa) patient cohorts. Supplementary Table 3. Primers, oligos, shRNAs, GapmeRs and probes used in the experiment. Supplementary Table 4. The list of lncRNAs that shared an overlap region with tumor related genes in retinoblastoma. Supplementary Table 5. Interactions between E2F3 and its targeting genes. Supplementary Table 6. Information of GO (Biological Process) Enrichment Analysis. Supplementary Table 7. ChIRP-MS identified lncRNA RBAT1 specifically binding proteins in Y79. Supplementary Table 8. ChIRP-MS identified lncRNA RBAT1 specifically binding proteins in 5637. Fig. S1. RBAT1 is a novel transcript in retinoblastoma (Rb) and bladder cancer (BCa). (A) Volcano plots of differentially expressed protein-coding genes. X axis represents log fold changes. Y axis represents log p values. Blue points denote significantly downregulated genes and red points denote significantly upregulated genes. (B) Agarose gel electrophoresis of PCR products generated by 3′ (left) and 5′ (right) RACE. (C) Schematic diagram of RACE assay. Fig. S2. RACE and coding potential analyses of RBAT1. (A) The full sequence of RBAT1. (B) Coding Potential Calculator (CPC) analysis suggested that RBAT1 is a noncoding RNA. GAPDH, CRTC1 and ACTB genes were used as protein-coding controls. XIST and HOTAIR genes were used as noncoding RNA controls. (C) RBAT1 was predicted by PhyloCSF to have no protein coding potential. The peaks showed the PhyloCSF score for each codon in each of 6 frames. Regions with a score less than 0 are predicted to be noncoding while Regions with a score greater than 0 are predicted to be coding. The protein coding gene ACTB and the noncoding RNA gene HOTAIR were used as controls. (D) Fractionation of tumor cell lines (Y79, WERI-Rb-1 and 5637) followed by RT-PCR. RBAT1 [file 12943_2020_1232_MOESM1_ESM.pdf]

Supplementary files

LncRNA RBAT1 accelerates tumorigenesis via interacting with HNRNPL and cis-activating E2F3

Xiaoyu He[1,2, *], Peiwei Chai[1,2, *], Fang Li[1,2, *], Leilei Zhang[1,2, *], Chuandi Zhou[1,2], Xiaolin Yuan[1,2], Yongyun Li[1,2], Jie Yang[1,2], Yingxiu Luo[1,2], Shengfang Ge[1,2], He Zhang[1,2, #], Renbing Jia[1,2, #], Xianqun Fan[1,2, #]

1. Department of Ophthalmology, Ninth People's Hospital, Shanghai JiaoTong University School of Medicine, Shanghai, China.
2. Shanghai Key Laboratory of Orbital Diseases and Ocular Oncology, Shanghai, China.

* These authors contributed equally to this report.

# These authors are co-corresponding and senior authors of this report.

Correspondence to: Xianqun Fan, **email**: fanxq@sjtu.edu.cn

Renbing Jia, **email**: renbingjia@sjtu.edu.cn

He Zhang, **email:** zhanghe@sjtu.edu.cn

Figure legend

Supplemental information includes 13 figures and 8 tables.

Supplementary Tables

Supplementary Table 1. Clinicopathological features and demographics of retinoblastoma (Rb) patient cohorts.

Supplementary Table 2. Clinicopathological features of Bladder Urothelial Carcinoma (BCa) patient cohorts.

Supplementary Table 3. Primers, oligos, shRNAs, GapmeRs and probes used in the experiment

Supplementary Table 4. The list of lncRNAs that shared an overlap region with tumor related genes in retinoblastoma.

Supplementary Table 5. Interactions between E2F3 and its targeting genes.

Supplementary Table 6. Information of GO (Biological Process) Enrichment Analysis.

Supplementary Table 7. ChIRP-MS identified lncRNA RBAT1 specifically binding proteins in Y79

Supplementary Table 8. ChIRP-MS identified lncRNA RBAT1 specifically binding proteins in 5637

Supplementary figure legends

Figure S1. RBAT1 is a novel transcript in retinoblastoma (Rb) and bladder cancer (BCa)

(**A**) Volcano plots of differentially expressed protein-coding genes. X axis represents log fold changes. Y axis represents log p values. Blue points denote significantly downregulated genes and red points denote significantly upregulated genes. (**B**) Agarose gel electrophoresis of PCR products generated by 3' (left) and 5' (right) RACE. (**C**) Schematic diagram of RACE assay.

Figure S2. RACE and coding potential analyses of RBAT1

(**A**) The full sequence of RBAT1. (**B**) Coding Potential Calculator (CPC) analysis suggested that RBAT1 is a noncoding RNA. GAPDH, CRTC1 and ACTB genes were used as protein-coding controls. XIST and HOTAIR genes were used as noncoding RNA controls. (**C**) RBAT1 was predicted by PhyloCSF to have no protein coding potential. The peaks showed the PhyloCSF score for each codon in each of 6 frames. Regions with a score less than 0 are predicted to be noncoding while Regions with a score greater than 0 are predicted to be coding. The protein coding gene ACTB and the noncoding RNA gene HOTAIR were used as controls. (**D**) Fractionation of tumor cell lines (Y79, WERI-Rb-1 and 5637) followed by RT-PCR. RBAT1 was mainly expressed in the nucleus. GAPDH and U6 RNA served as positive controls for the cytoplasmic and nuclear fractions, respectively.

Figure S3. Effect of RBAT1 on colony formation ability and migration ability of tumor cell lines

(A) A colony formation assay was performed to determine the colony formation ability of RBAT1-silenced tumor cell lines (Y79, WERI-Rb-1 and 5637). For colony formation assays, 500 cells were seeded in 6-well plates (Poly-L-lysine-coated 6-well plates for retinoblastoma

cell lines). 7-14 days later, the colonies were washed with PBS, fixed and stained for 20 min with a 1% crystal violet solution. Images were captured by a scanner, and the percentage of cell occupancy was counted and analyzed by ImageJ software. (**B**) The migration and invasion abilities displayed no significant changes in RBAT1-silenced tumor cell lines (5637) compared with ctrl group. (**C**) A colony formation assay was performed to test sustained effect after removing GapmeRs at the 3rd day. (**D**) A real-time PCR was performed to determine the expression level of RBAT1 in tumors from GapmeR1/2 treated groups and Ctrl. The results are shown as the mean ± SD in three independent experiments. *p <0.05 and **p <0.01.

Figure S4. Gene expression patterns associated with cell proliferation in RBAT1-silenced Rb and BCa cell lines.

(**A**) Gene expression profiles in 5637 cells and WERI-Rb-1 cells transfected with GapmeR1/2 or a ctrl GapmeR. (**B**) The interaction of genes in cell cycle pathway. The down-regulated genes in red semitransparent area were verified in our study.

Figure S5. The overexpression of RBAT1 and E2F3 in normal cell lines

(**A**) Realtime-PCR showed the expression levels of RBAT1 after transfecting pcDNA3.1-RBAT1 in ARPE-19 and SV-HUC-1. (**B-C**) Western blot was performed to test E2F3 expression after transfecting pcDNA3.1-RBAT1 or pcDNA3.1-E2F3 in ARPE-19 and SV-HUC-1, respectively. (**D-E**) Colony formation assays were performed to measure the colony formation ability of ARPE-19 and SV-HUC-1 with RBAT1 or E2F3 overexpression. (**F**) CCK8 assay was performed to assess cell proliferation of RBAT1 or E2F3 over-expressed normal cell lines (ARPE-19 and SV-HUC-1). The results were shown as Mean ± SD, *p <0.05; **p <0.01.

Figure S6. ChIRP-MS analysis of RBAT1-interacting proteins.

The protein peptides isolated by ChIRP. U6 was selected as control, and scrambled oligos were

selected as negative controls.

Figure S7. HNRNPL expression analysis

(**A**) Western blot analysis showed that RBAT1 depletion did not influence the expression level of HNRNPL. (**B**) HNRNPL was silenced in tumor cell lines (Y79, WERI-Rb-1 and 5637) by two independent shRNAs.

Figure S8. Alignment of RBAT1 sequences and human genome sequences.

lncRNA RBAT1 could not only align to E2F3 promoter (chr6:20401729-20481937, 100% similarity, 1st panel), but also 5kb upstream from TSS of SLFN12L (chr17:35490212-35490325, 94.8% similarity, 2nd panel) and an intronic region of DNAH7 (chr2:195995428-195995503, 87.1% similarity, 3rd panel).

Figure S9. Identification of the E2F3 promoter region

(**A**) Schematic of the luciferase assay of E2F3 promoter. 2000bp upstream to E2F3 TSS was selected for detection. The full-length 5' to 3' sequence was used as positive control (lane a). The untreated group (NC) and empty vector group (Mock) served as negative control groups. Different segments of E2F3 promoter (lane b-e) were constructed into pGL3 vector and subjected to luciferase reporter assays in HEK-293T cells. Through luciferase reporter assays, we identified a -500 to -1000 bp segment of TSS upstream region as the core promoter of E2F3 with transcriptional activity.

Figure S10. Copy number variation (CNV) analysis of Rb cell lines.

(**A** and **B**) CNV microarray showed increased E2F3 gene copy number in Y79 and WERI-Rb-1 compared with ARPE-19. RBAT1 interference did not influence the CNV of retinoblastoma

cell lines. The Affymetrix OncoScan CNV microarray was used to detect the CNV of cells. The arrays were scanned with a GeneChip Scanner 3000 7G System and the data were analyzed using Chromosome Analysis Suite (ChAS).

Figure S11. Copy number variation (CNV) analysis of BCa cell lines.

(**A** and **B**) CNV microarray of bladder cancer cell line showed increased E2F3 gene copy number in 5637 compared with SV-HUC-1. RBAT1 silencing did not influence the CNV of bladder cancer cell lines.

Figure S12. HT1376, a bladder cancer cell line without E2F3 copy number amplification, represent highly-expressed RBAT1.

(A) HT-1376, a bladder cancer cell line without E2F3 locus amplification. **(B)** Real-time PCR showed that RBAT1 was also highly expressed in HT-1376. **(C)** Real-time PCR showed that RBAT1 was knockdown by GapmeR1. (**D** and **E**) After RBAT1 knockdown the expression of E2F3 was largely reduced **(D)** and the tumor proliferation was also suppressed **(E).** The results were shown as Mean ± SD, $^{**}p < 0.01$.

Figure S13. E2F3 associates with Rb and BCa clinical characteristics and predict disease prognosis.

(**A**) Box plot represented E2F3 expression in Rb (left) and BCa (right) specimens at stage I (Rb: n=5; BCa: n=6) and at stage II/III/IV (Rb: n=15; BCa: n=19). $^{*}p < 0.05$; $^{**}p < 0.01$. (**B**) Kaplan-Meier survival analysis was performed in 224 cases of bladder cancer using R2 platform (https://hgserver1.amc.nl). (**C**)The normalized expression value of E2F3 in 224 BCa patients were automatically calculated in R2 platform.

Supplementary Figure 1

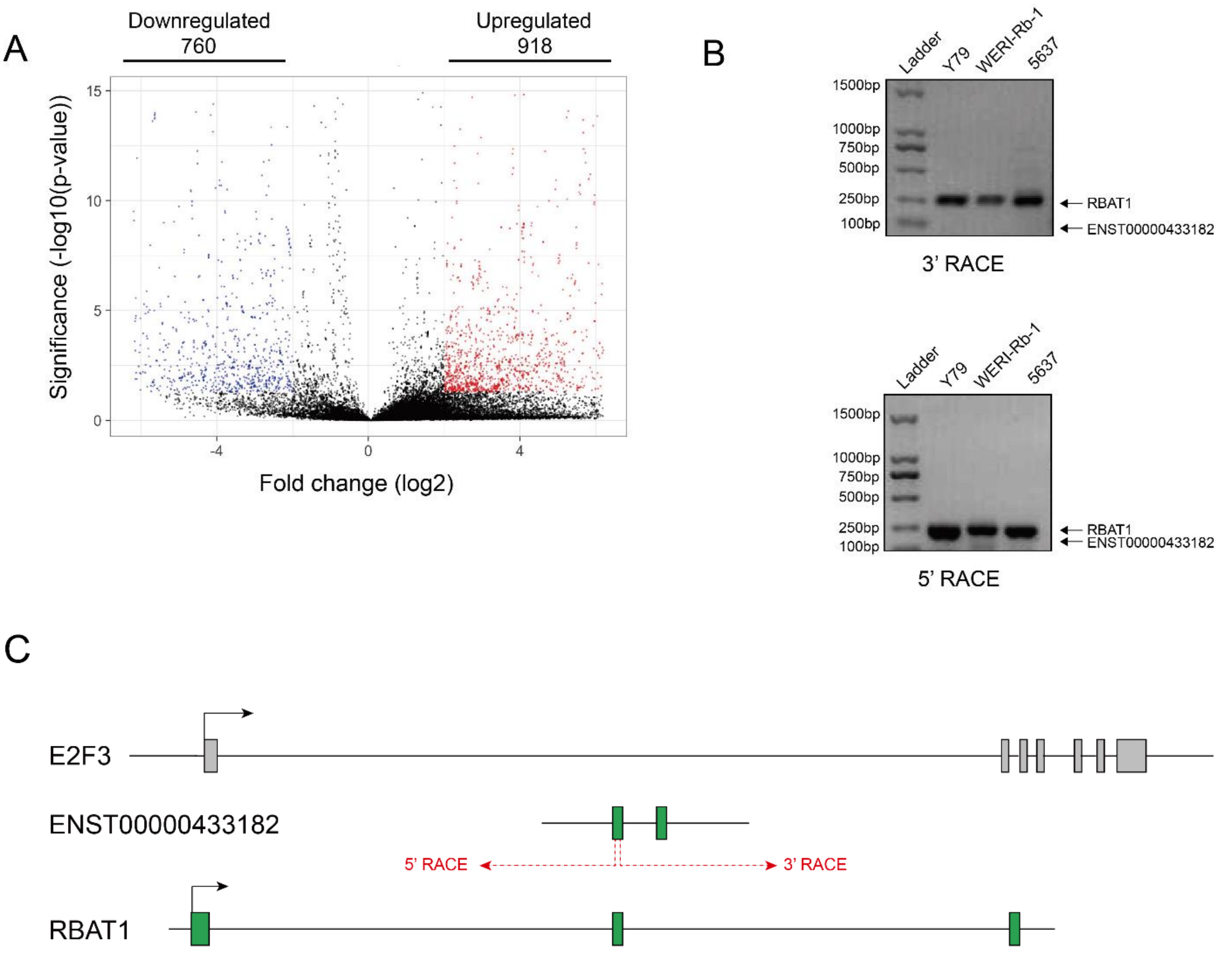

Supplementary Figure 2

A

RBAT1 Full length Sequence:

TTGGGACTGAAGTAAGGAGGTAGCGGCCCAATGGGCTGTGGGAACGGTCCTCGGCGGGTTGAGGGGCGGG
GATATGCAAATATGGTTTGAAAAGCCGGCGGGAAATCCGAGTTTCGCGGGAGGACCTTGGCGCGTAAACCGT
ATCCCTTCATTCATTGTCAGCAGCAGCTTCCTGGAGCCATTTTTCAGCTGCCGGCCGCAGCACCCGGGAAGC
AGAGTCCAAGAGAAATGCCCTGCCCAGGGTCTGGCAGAACTGATGGAGAGCAGCCTTCGTGGCTACCACCT
TCCCATAAAGTCTGTGCTCTCCAGCGGCGCTTGGCAAAGCGAAGGCTGGAGCTAGGAGAAAGCGGTCATCA
GTACCTCTCAGATGGTTTAAAAACCCCCAAGGGCAAAGGAAGAGCTGCACTACGAAGTCCAGATAGTCCAAAA
AGTAAGGATCTTTTCATCTCTTTCCTTATTCTCCTTGGTATGGCATTTCCAAGTTTCAAAGCTTATGGCCGGAAG
GATGCCAAGGTGTGAATAATTCTGGCATGTTTTGTTCTTTTTCATTTCTTTCTGTGCTTATCAAAAAAAAAAAAAA
AA

C

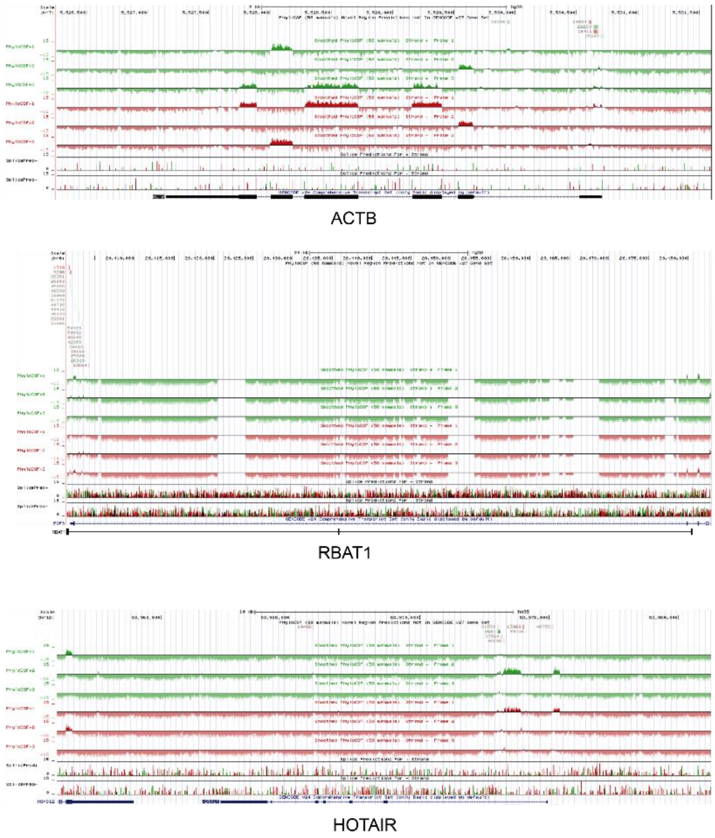

B

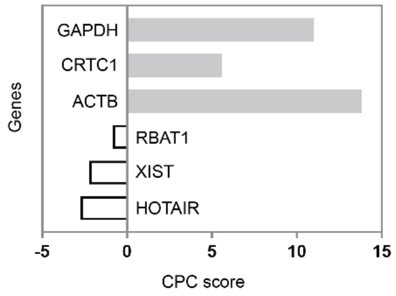

D

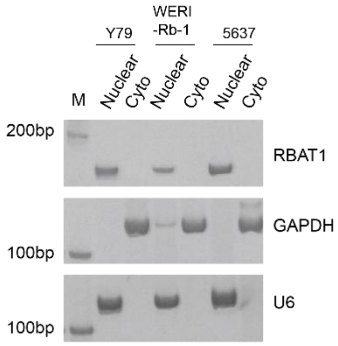

Supplementary Figure 3

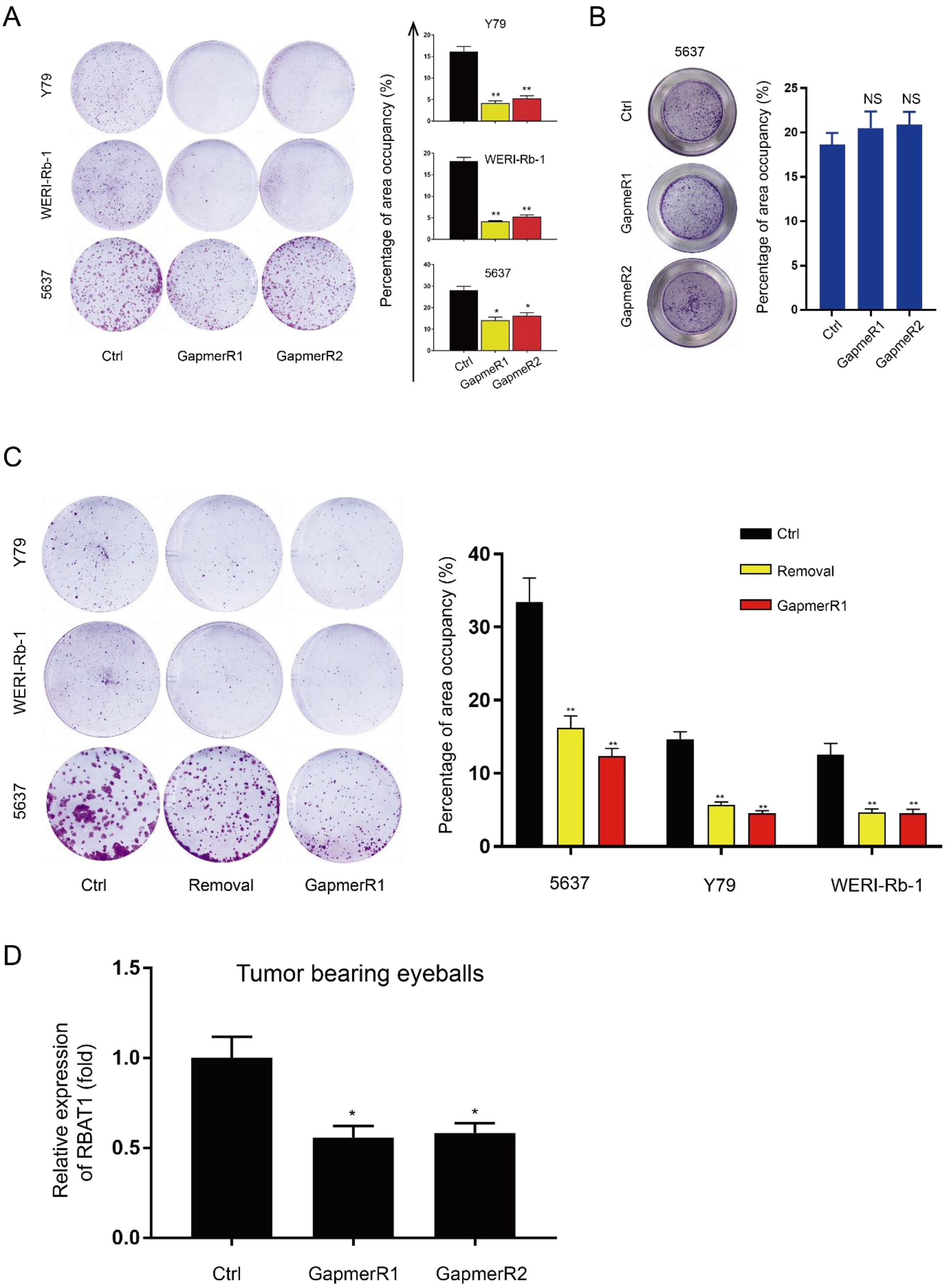

Supplementary Figure 4

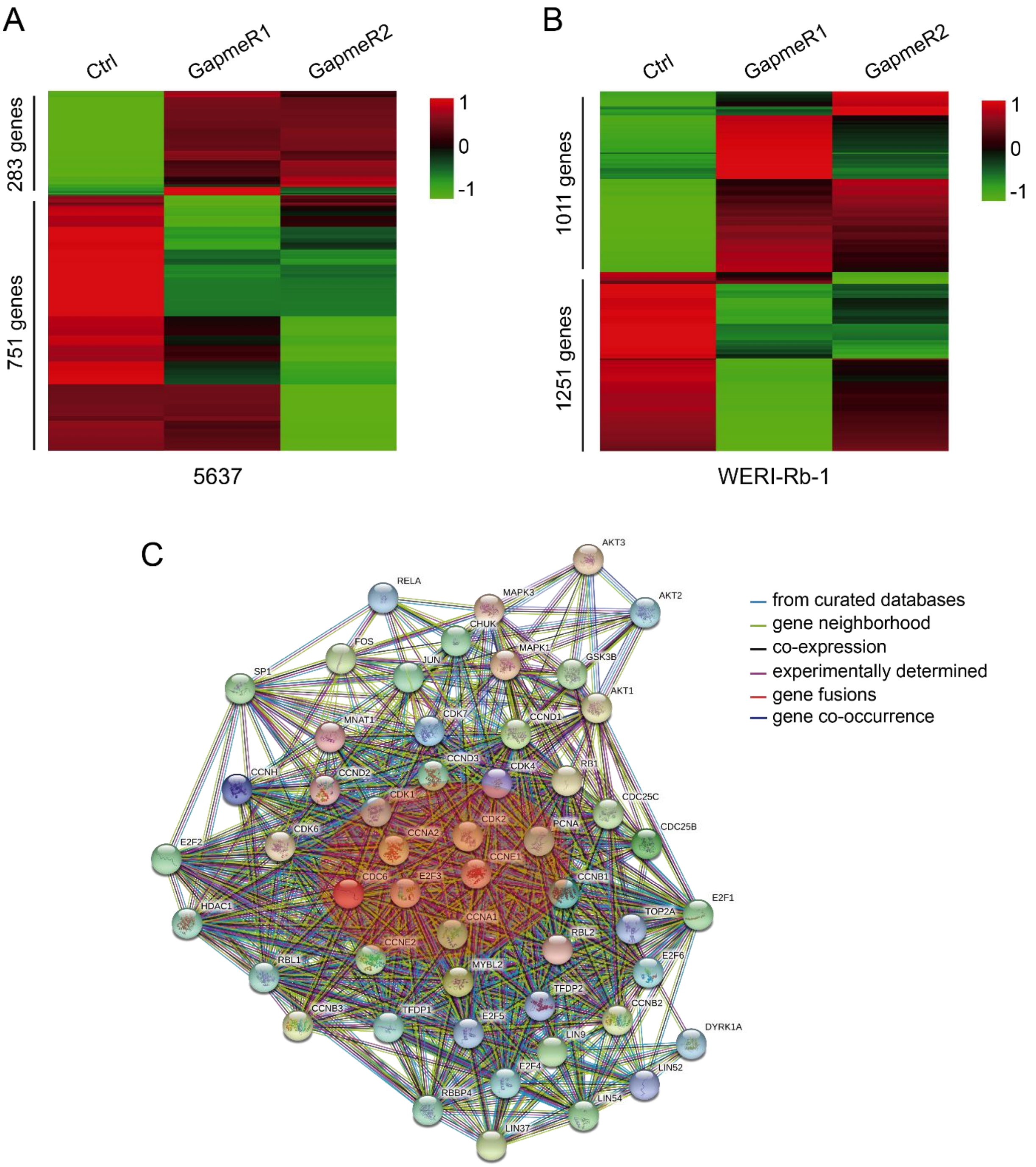

Supplementary Figure 5

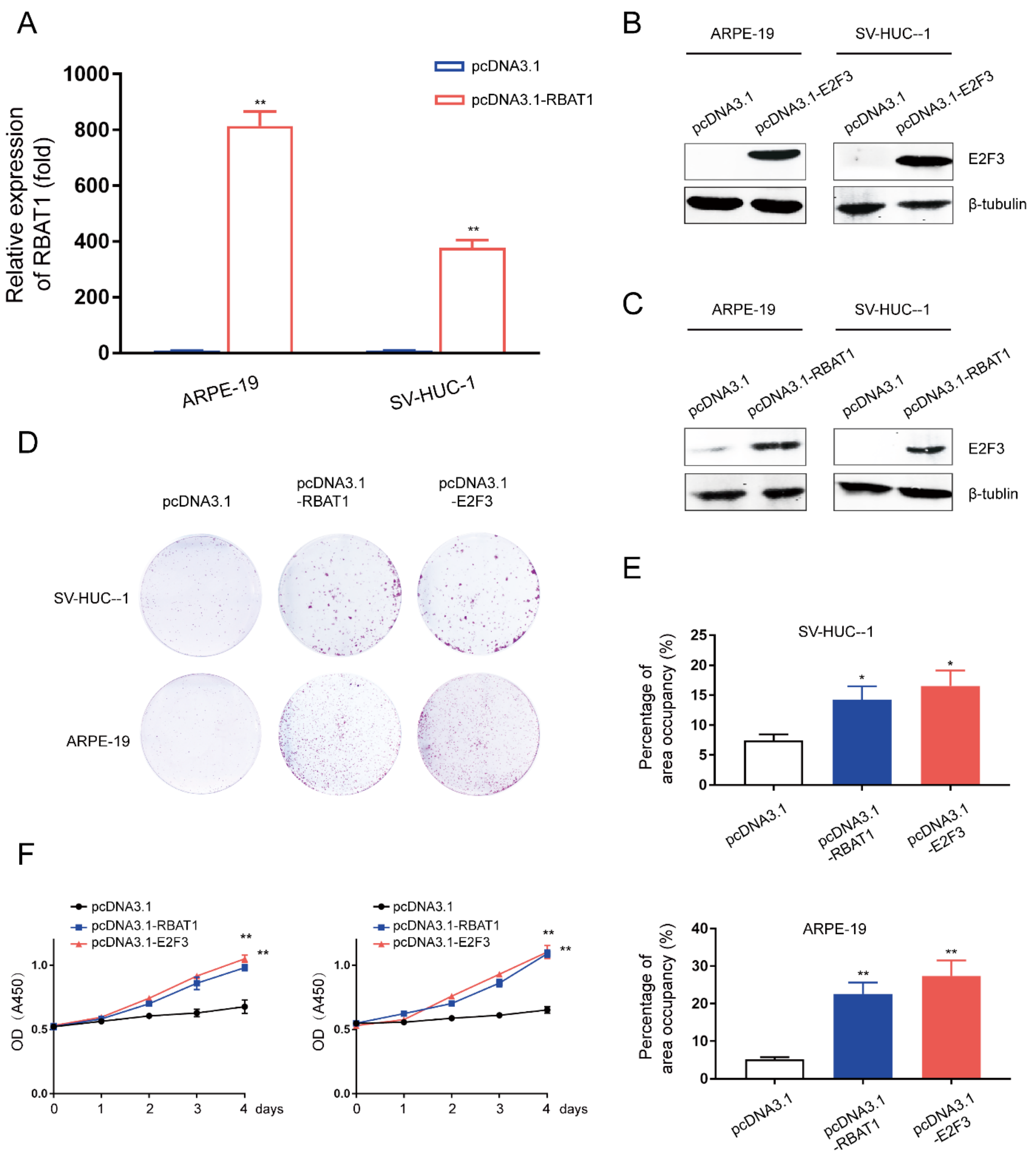

Supplementary Figure 6

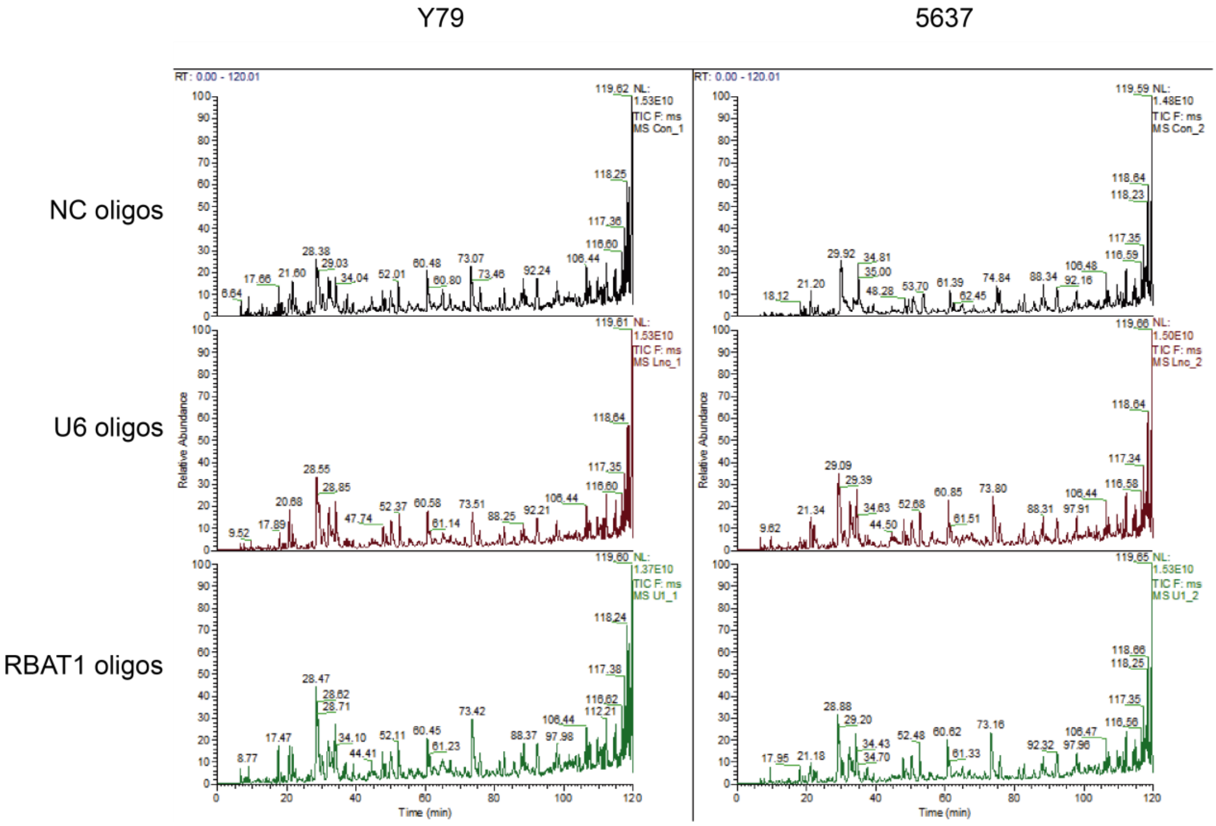

Supplementary Figure 7

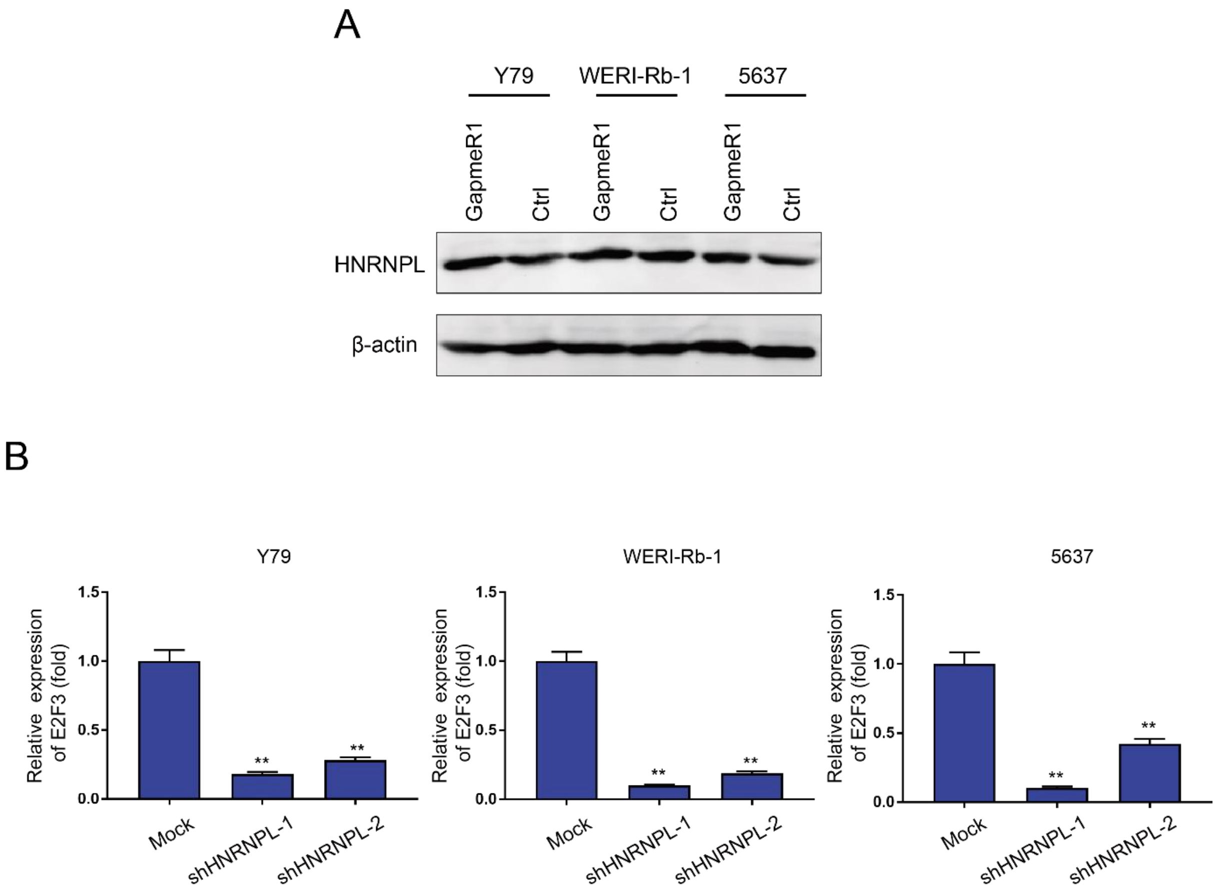

Supplementary Figure 8

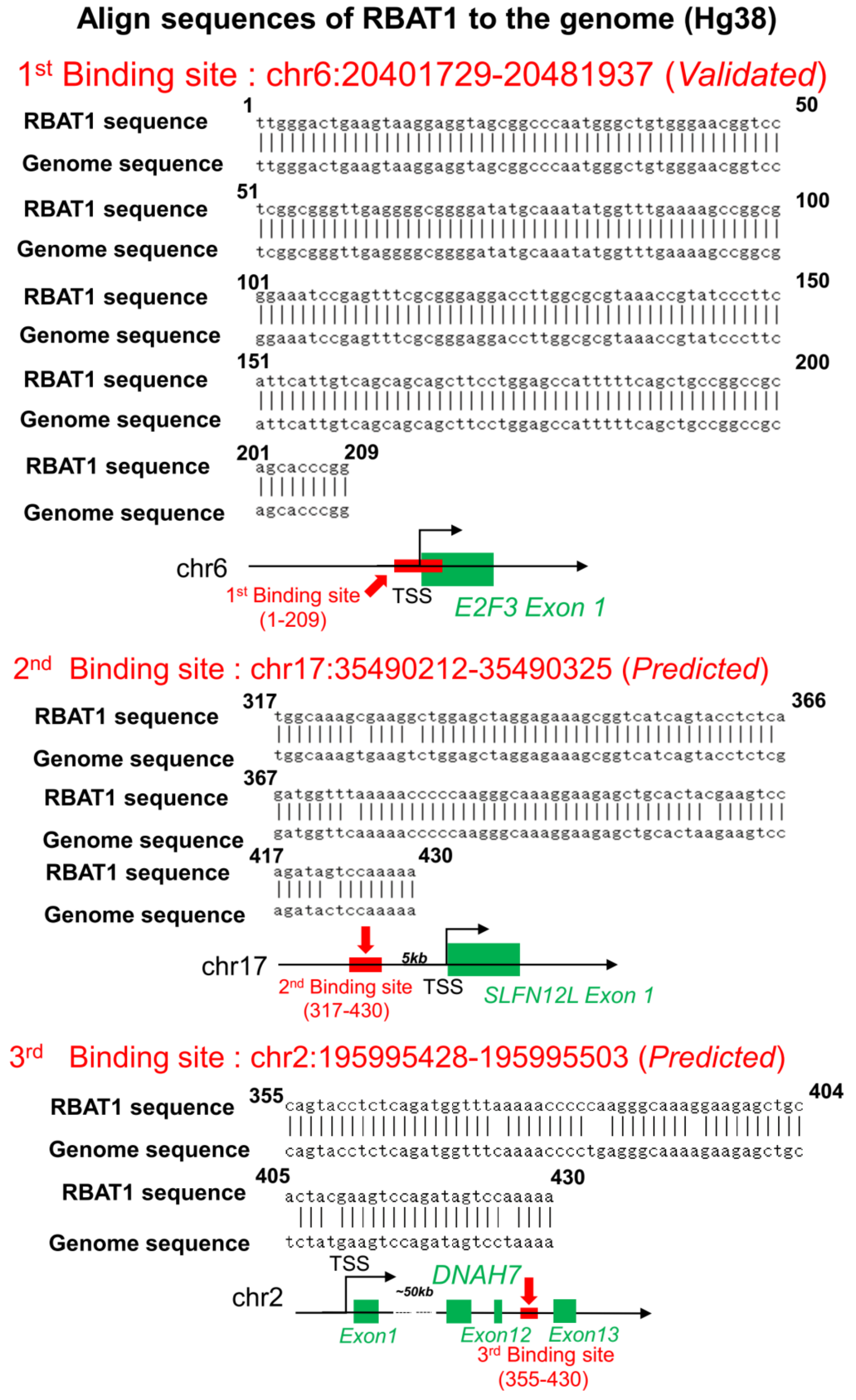

Supplementary Figure 9

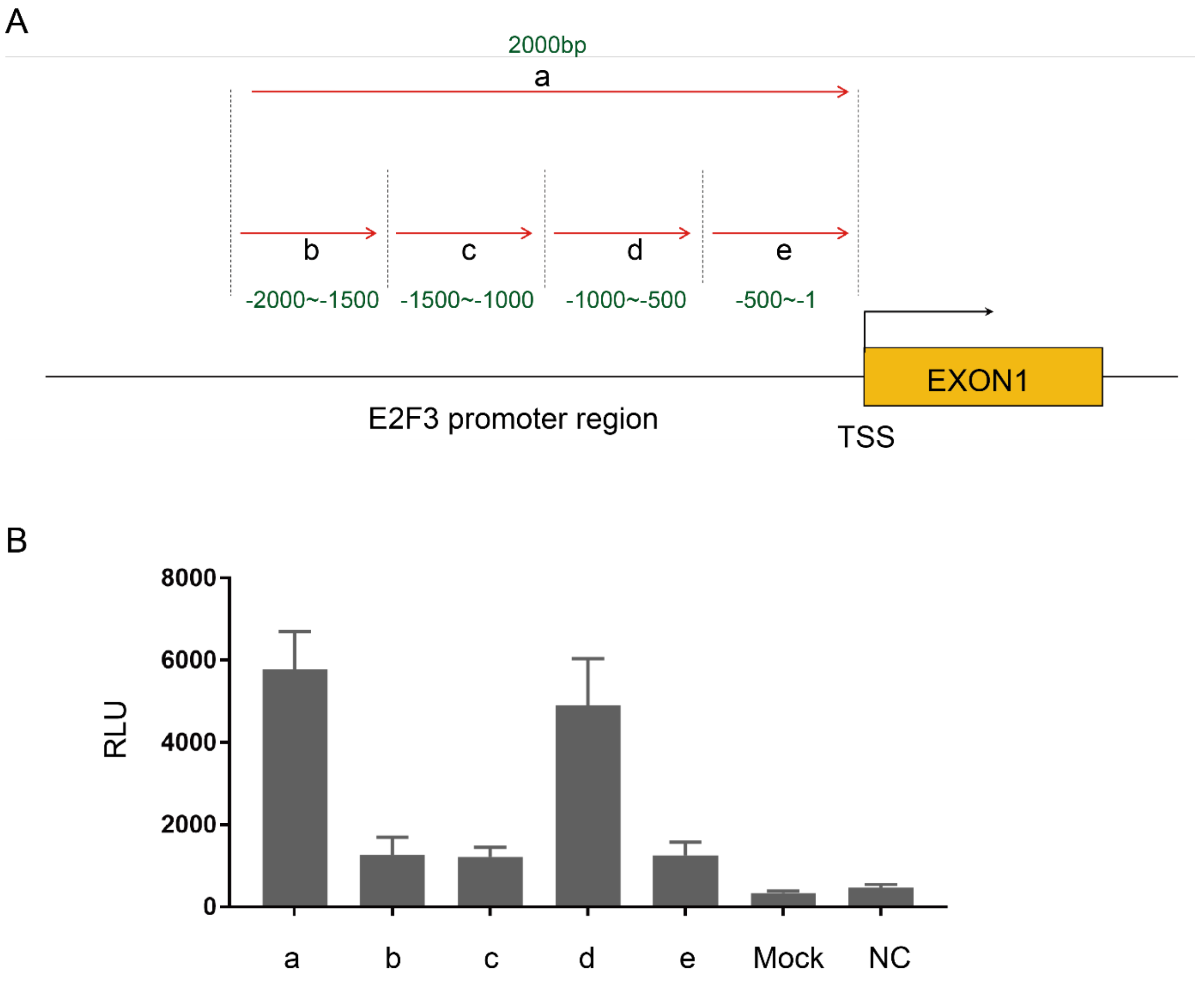

Supplementary Figure 10

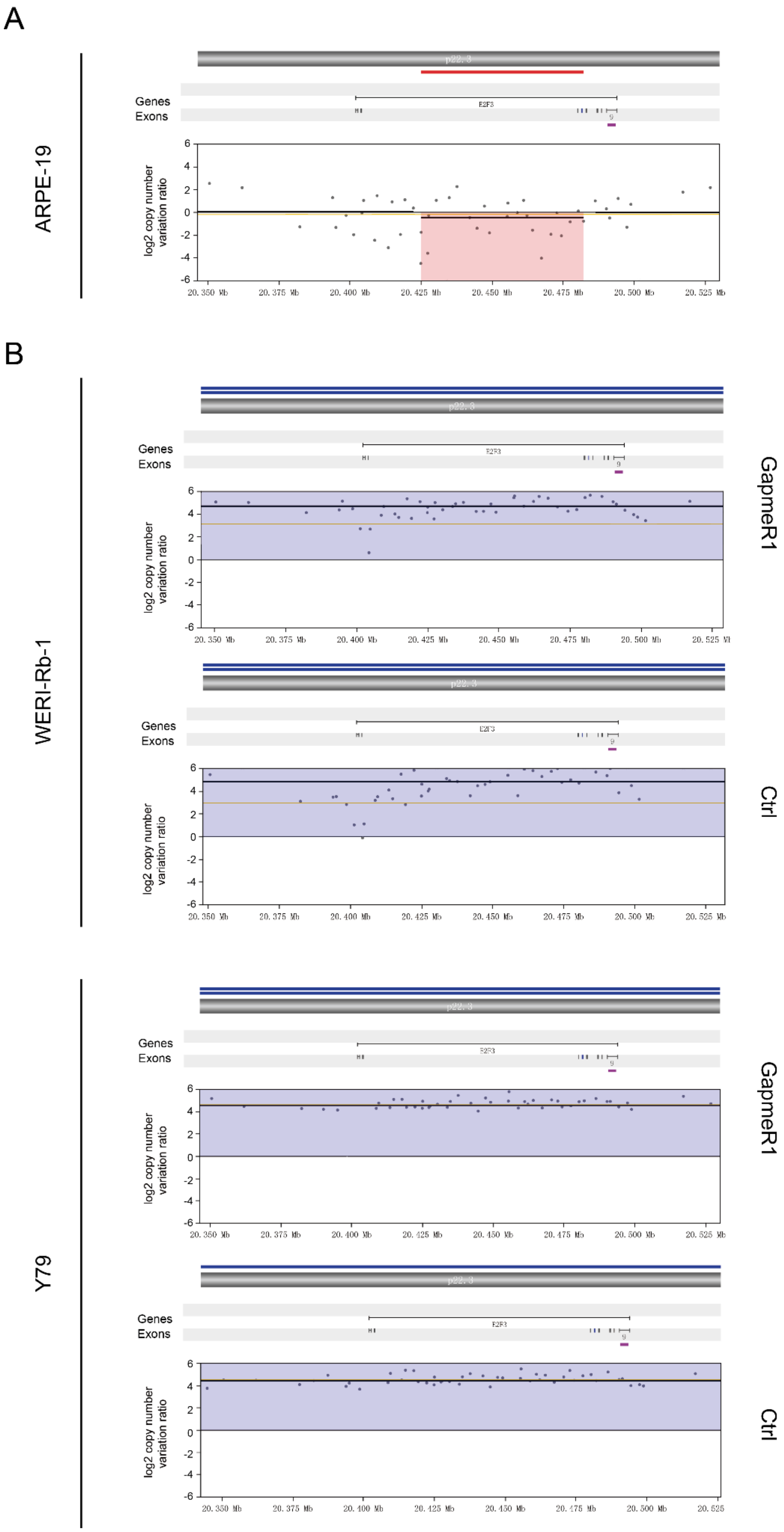

Supplementary Figure 11

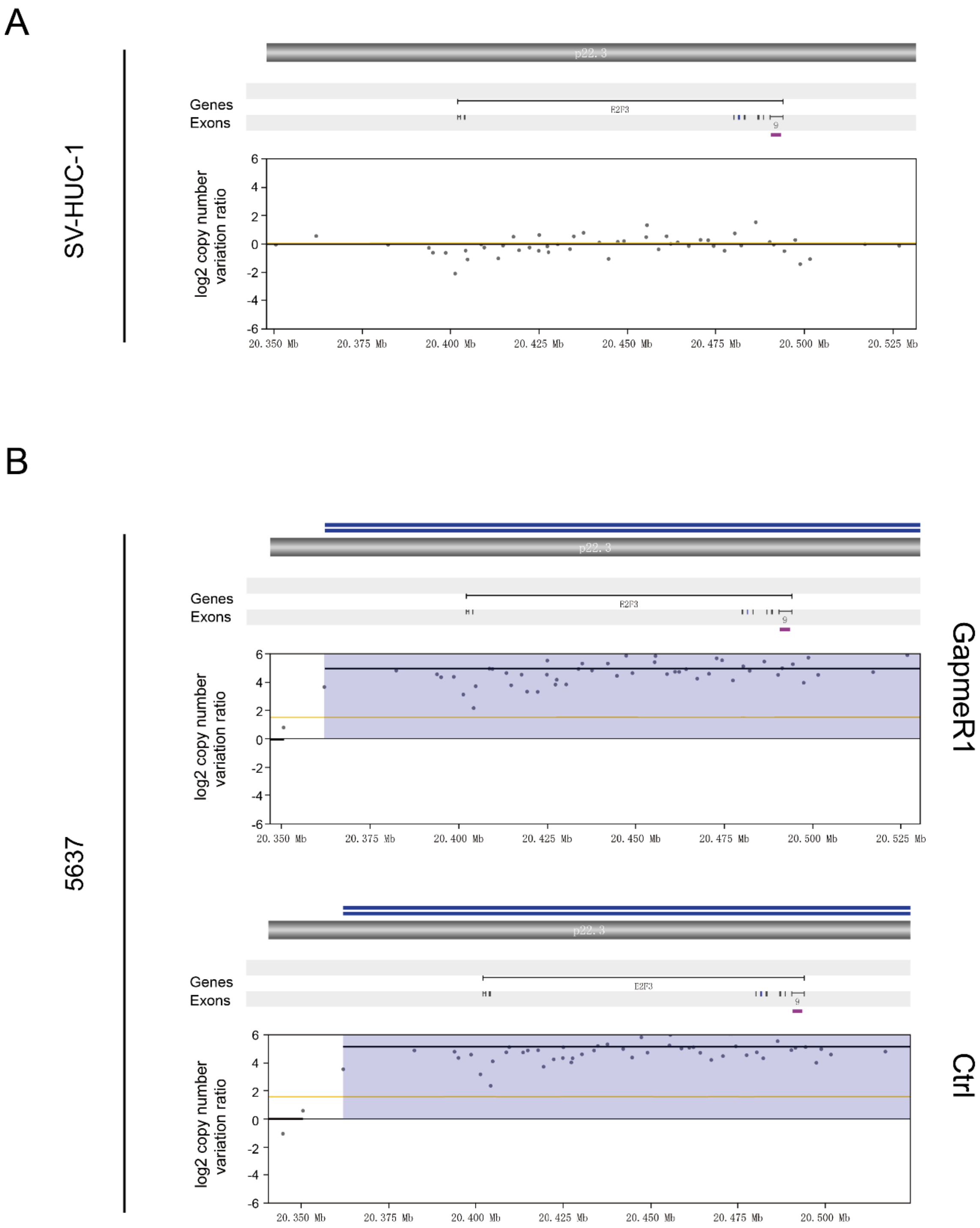

Supplementary Figure 12

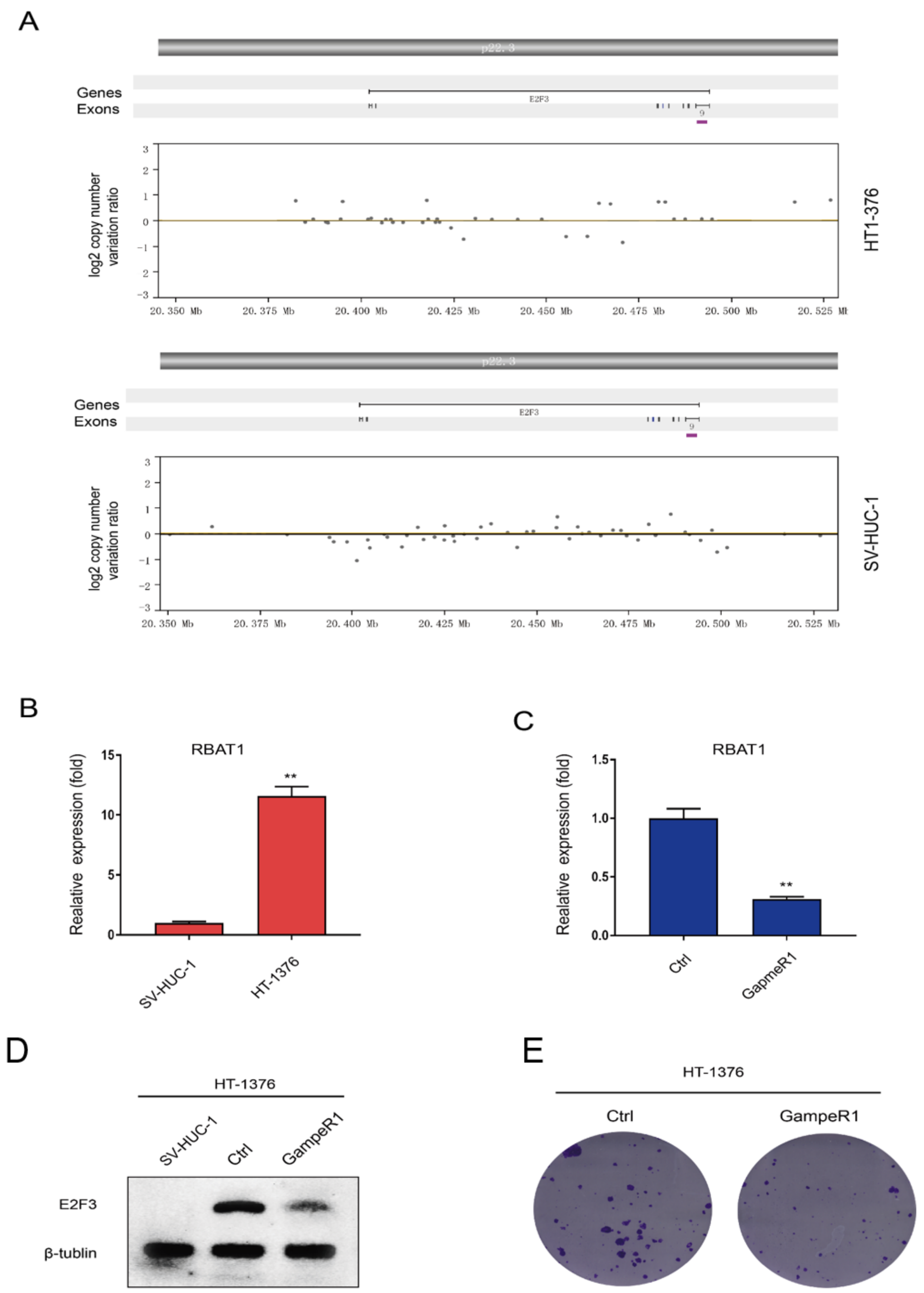

Supplementary Figure 13

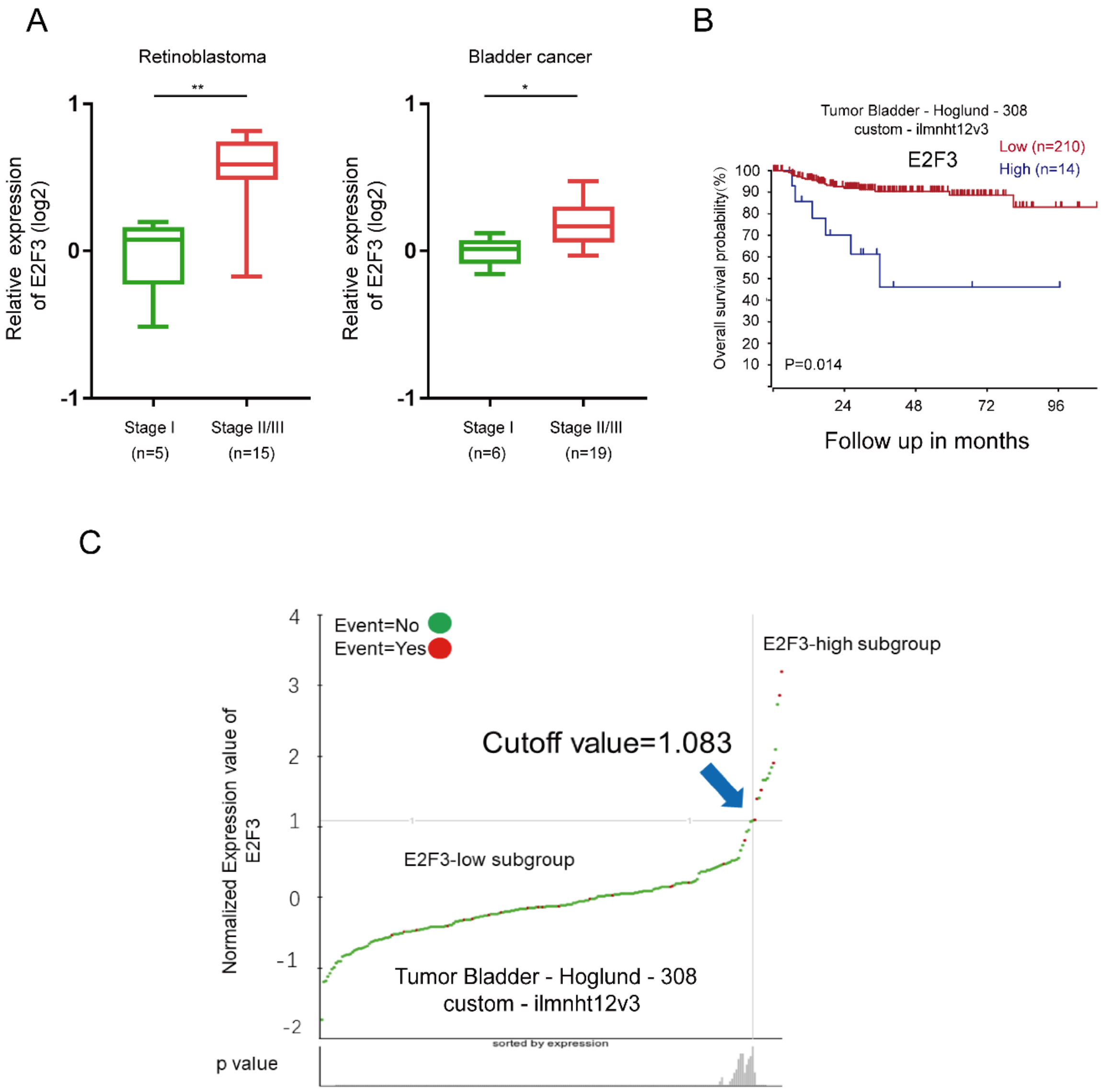

Supplementary Table 1. Clinicopathological features and demographics of retinoblastoma (Rb) patient cohorts.

**Pathology diagnosis**	**Age**	**Sex**	**Stage**
Rb	2	F	I
Rb	1	F	I
Rb	3	M	I
Rb	2	M	II
Rb (optic nerve invasion)	3	M	III
Rb	2	M	I
Rb	8 Mon	M	II
Rb (optic nerve invasion)	4	M	II
Rb (necrosis)	1	F	II
Rb (optic nerve invasion)	6	F	II
Rb	2	M	II
Rb	1	F	II
Rb	5	F	II
Rb	2	M	I
Rb	4	M	II
Rb	2	M	I
Rb	9 Mon	M	II
Rb (optic nerve invasion)	2	F	III
Rb	2	F	II
Rb (necrosis)	8	M	II
Adjacent normal retina tissue	1	F	
Adjacent normal retina tissue	2	M	
Adjacent normal retina tissue	11	F	
Adjacent normal retina tissue	4	M	
Adjacent normal retina tissue	5	F	
Adjacent normal retina tissue	2	F	

Supplementary Table 2. Clinicopathological features of Bladder cancer (BCa) patient cohorts.

Pathology diagnosis	Age	Sex	Stage
BCa	37	F	I
BCa	65	M	II
BCa	54	F	II
BCa	72	M	III
BCa (squamous metaplasia)	62	F	III
BCa	60	M	I
BCa	53	M	II
BCa	65	M	II
BCa	56	M	I
BCa	58	M	I
BCa	48	M	II
BCa	50	M	II
BCa	52	F	I
BCa	64	M	III
BCa (necrosis)	49	M	III
BCa	42	M	II
BCa	52	M	II
BCa	45	M	III
BCa	44	M	I
BCa	50	F	II
BCa	42	M	II
BCa	63	F	II
BCa	43	F	II
BCa	61	M	II
BCa	51	M	II
Adjacent normal bladder tissue	54	M	
Adjacent normal bladder tissue	40	M	
Adjacent normal bladder tissue	57	F	
Bladder tissue	50	M	
Bladder tissue	48	F	

Supplementary Table 3. Primers, oligos, shRNAs, GapmeRs and probes used in the experiment

RT-PCR primers:

Gene	Forward primer	Reverse primer	Amplicon (bp)
RBAT1	GAAGGCTGGAGCTAGGAGAA	TAGTGCAGCTCTTCCTTTGCCC	83
E2F3	TTTGGCAAGTACCCAAGGGC	GGAGGCCAGAGGAGAAAGGTT	193
GAPDH	CAATGACCCCTTCATTGACC	GACAAGCTTCCCGTTCTCAG	106
AURKA	GGAATATGCACCACTTGGAACA	TAAGACAGGGCATTTGCCAAT	108
CCNE1	GCCAGCCTTGGGACAATAATG	CTTGCACGTTGAGTTTGGGT	104
CCNA1	GAGGTCCCGATGCTTGTCAG	GTTAGCAGCCCTAGCACTGTC	82
CCNA2	CGCTGGCGGTACTGAAGTC	GAGGAACGGTGACATGCTCAT	120
CDK1	AAACTACAGGTCAAGTGGTAGCC	TCCTGCATAAGCACATCCTGA	148
CDC6	CCAGGCACAGGCTACAATCAG	AACAGGTTACGGTTTGGACATT	116
PCNA	CCTGCTGGGATATTAGCTCCA	CAGCGGTAGGTGTCGAAGC	109
DHFR	ACAATGGAACCATGCCTTCA	TGTACTCCTTCATTTTGCTGG	208
CDK2	CCAGGAGTTACTTCTATGCCTGA	TTCATCCAGGGGAGGTACAAC	90
U6	AAAGCAAATCATCGGACGACC	GTACAACACATTGTTTCCTCGGA	181
MBOAT1	TGTGCTGGTGTTAATGTGCTAT	GGCTGATGTGGCATATTGTAAGA	110
CDKAL1	GGGACTGAGTATCATTGGGGT	CCAAGCCGCCTTCCATTATC	120
THRIL	AACTCCTGACCTCAGGTGATCCAT	AAGGGAGTTTCAGAAGGTGTGGCT	161

CHIP-PCR primers:

Promoter	Forward primer	Reverse primer	Amplicon (bp)
E2F3(P1-P2)	AATCGCGTCTGCTGAACCTTC	TACGTGCCAAAGGCAAGTTG	193
E2F3(P3-P4)	TTAGACCTGAGTGCTCTTCCC	GTTTGATGTGGTTCGCGCCA	96
E2F3(P5-P6)	TGGTCAGTAGGGGTATAGGCT	TGAGTTTACGTCTGCCGCCTC	159
GAPDH	AGAAAGAAAGGGGAGGGGGCA	AGCAGGACACTAGGGAGTCAAG	146

Primers for RBAT1 RACE:

Primers for 5' RACE:	Primers for 3' RACE:
GCCCTGCCCAGGGTCTGGCAGAACTGATG	CCGCTGGAGAGCACAGACTTTATGGGAA

Stellaris probes for RNA-FISH:

RBAT1	CTATCTGGACTTCGTAGTGC
U6	GCTTTAAGAAGTCACTAGGGGT
GAPDH	CACTTCCCTTGTATCGGCCGT

ChIRP probes for RBAT1:

RBAT1	CCTTCACATCAAGCTGATGA
	CCATCTGAGAGGTACTGATG

Sequences of GampeRs:

GapmeR1:	AGGGCATTTCTCTTG
GapmeR2:	GCACAGACTTTATGGG
Non-targeting GapmeR:	TCGTAGTGCAGCTCT

shRNA Designs:

E2F3-1	GGAGAUAGUCCUCUUGCAG
E2F3-2	GACUUAGCCAGGAGAUCUU
HNRNPL-1	CUUCUGGCUGGUAGAGUAG
HNRNPL-2	AUUCACACCUUGGCAUCC

Supplementary Table 4. lncRNAs that shared an overlap region with tumor related genes in retinoblastoma

Gene	log2(Fold_change)	Transcript ID	strand	transcript size	log2(Fold_change of lncRNA)
MYCN	3.696	ENST00000419083	-	770	3.688
E2F1	3.648	ENST00000606866	+	440	3.8
E2F2	3.215	N. A	N. A	N. A	N. A
E2F3	2.874	ENST00000433182	+	582	4.898
CDH13	-4.456	NR_033984	+	2005	1.531
		ENST00000565238	+	1493	-2.456
		ENST00000567359	+	4037	-1.849
		ENST00000563342	-	702	-3.554
		ENST00000563981	-	906	-3.147
		ENST00000569165	-	686	-1.721
		ENST00000570056	-	544	-1.878
		ENST00000567860	-	745	-1.775
		ENST00000562565	-	563	-1.952
		ENST00000564635	-	403	-1.817
CCNA2	1.727	N. A	N. A	N. A	N. A
CDK1	1.551	N. A	N. A	N. A	N. A
CCNE1	2.933	N. A	N. A	N. A	N. A
PCNA	2.778	N. A	N. A	N. A	N. A
DHFR	2.483	ENST00000514201	+	549	2.785

Supplementary Table 5. Interactions between E2F3 and its targeting genes.

Gene	Targeting gene	Targeting gene accession	Score
E2F3	CDK1	ENSP00000378699	0.964
E2F3	CCNE1	ENSP00000262643	0.974
E2F3	CCNA1	ENSP00000255465	0.977
E2F3	CCNA2	ENSP00000274026	0.988
E2F3	CDC6	ENSP00000209728	0.962
E2F3	PCNA	ENSP00000368438	0.834
E2F3	DHFR	ENSP00000396308	0.506
E2F3	CDK2	ENSP00000266970	0.975
E2F3	MYBL2	ENSP00000217026	0.830
E2F3	VEGFA	ENSP00000361125	0.534
E2F3	CDKNA1	ENSP00000244741	0.741
E2F3	CDC25A	ENSP00000303706	0.504
E2F3	CCNB2	ENSP00000288207	0.665
E2F3	AURKA	ENSP00000216911	0.412

Supplementary Table 6. Information of GO (Biological Process) Enrichment Analysis.

Term ID	Term description	Fold enrichment	P_value
GO:0006335	mitotic cell cycle phase transition	7.201207822	1.01E-11
GO:0007067	mitotic nuclear division	8.283742331	2.28E-08
GO:0051301	cell division	7.10363048	5.07E-07
GO:0006260	DNA replication	5.710310279	1.17E-06
GO:0071157	regulation of cell cycle arrest	4.267382413	1.94E-06
GO:0000082	G1/S transition of mitotic cell cycle	7.098220251	2.10E-06
GO:0051726	regulation of cell cycle	7.612087548	2.51E-05
GO:0007062	sister chromatid cohesion	4.864815951	2.52E-04
GO:0045814	DNA replication initiation	6.270439464	2.57E-04

Supplementary Table 7. ChIRP-MS identified lncRNA RBAT1 specifically binding proteins in Y79

Gene	iBAQ	Unique. peptides RBAT1	Unique. peptides NC	Rank	Score
HRNR	33128000	14	2	20	323.31
RPL34	27184000	5	0	24	8.4139
NOP56	691730	6	0	203	8.5572
HNRNPL	7244500	7	0	83	7.9645
RPL32	23277000	5	0	30	9.6512
RPL7A	6983200	5	0	87	5.2823
XP32	5322500	6	0	103	6.3086
SERPINB12	4210300	5	0	111	5.8999
CAT	1876100	4	0	147	8.8183
LOR	16684000	3	0	42	5.5403
RPL6	16182000	13	0	43	27.374
DSC1	6672400	8	1	91	21.363
KRT78	13576000	18	1	49	53.396
RPL8	25478000	7	0	25	12.325
RPL18	52119000	6	0	12	36.647
DSG1	7066200	19	0	84	79.347
RPS9	18752000	7	0	37	8.8857

Supplementary Table 8. ChIRP-MS identified lncRNA RBAT1 specifically binding proteins in 5637

Gene	iBAQ	Unique. peptides RBAT1	Unique. peptides NC	Rank	Score
RPL4	10182000	6	2	59	9.3873
FABP5	8785100	5	2	69	16.125
RPL13	19249000	9	4	36	45.802
CSTA	10956000	4	1	57	5.883
RPS4X	12002000	6	1	53	4.5523
RPS6	19275000	7	2	35	38.341
HNRNPL	7244500	7	0	33	7.9645
HNRNPK	9734200	14	0	64	54.765
CCAR2	188480	7	0	266	5.6181
